# CRLF1 bridges AKT and mTORC2 through SIN1 to inhibit pyroptosis and enhance chemo-resistance in ovarian cancer

**DOI:** 10.1038/s41419-024-07035-4

**Published:** 2024-09-10

**Authors:** Cong Xiang, Li Chen, Shilei Zhu, Yue Chen, Haodong Huang, Chunmao Yang, Yugang Chi, Yanzhou Wang, Yunlong Lei, Xiongwei Cai

**Affiliations:** 1 Department of Obstetrics and Gynecology, Chongqing Health Center for Women and Children (Women and Children’s Hospital of Chongqing Medical University), Chongqing, China; 2 Life Sciences Institute, Zhejiang University, Hangzhou, Zhejiang China; 3 Department of Gynecology, Southwest Hospital, Chongqing, China; 4 Department of Biochemistry and Molecular Biology, College of Basic Medical Sciences, Chongqing Medical University, Chongqing, China; 5 Molecular Medicine and Cancer Research Center, College of Basic Medical Sciences, Chongqing Medical University, Chongqing, China

**Keywords:** Diagnostic markers, Chemotherapy

## Abstract

Ovarian cancer, the second most leading cause of gynecologic cancer mortality worldwide, is challenged by chemotherapy resistance, presenting a significant hurdle. Pyroptosis, an inflammation-linked programmed cell death mediated by gasdermins, has been shown to impact chemoresistance when dysregulated. However, the mechanisms connecting pyroptosis to chemotherapy resistance in ovarian cancer are unclear. We found that cytokine receptor-like factor 1 (CRLF1) is a novel component of mTORC2, enhancing AKT Ser473 phosphorylation through strengthening the interaction between AKT and stress-activated protein kinase interacting protein 1 (SIN1), which in turn inhibits the mitogen-activated protein kinase kinase kinase 5 (ASK1)-JNK-caspase-3-gasdermin E pyroptotic pathway and ultimately confers chemoresistance. High CRLF1-expressing tumors showed sensitivity to AKT inhibition but tolerance to cisplatin. Remarkably, overexpression of binding-defective CRLF1 variants impaired AKT-SIN1 interaction, promoting pyroptosis and chemosensitization. Thus, CRLF1 critically regulates chemoresistance in ovarian cancer by modulating AKT/SIN1-dependent pyroptosis. Binding-defective CRLF1 variants could be developed as tumor-specific polypeptide drugs to enhance chemotherapy for ovarian cancer.

## Introduction

Ovarian cancer (OC) is an aggressive gynecological malignancy with a poor prognosis. Despite progress in treatment, the overall survival rate remains below 40% [1, 2]. Current treatment predominantly revolves around debulking surgery followed with platinum-based chemotherapy, such as cisplatin [3, 4]. However, chemotherapy resistance is a major challenge leading to treatment failure [3, 5, 6]. Enhancing cancer cell susceptibility to programmed cell death induced by chemotherapeutic drugs is crucial to overcome this resistance.

Pyroptosis, a form of inflammatory cell death mediated by gasdermins (GSDMs), has emerged as a potential strategy to overcome chemotherapy resistance [7–11]. One key gasdermin, GSDME, shifts cell death from apoptosis to pyroptosis in response to chemotherapy drugs, thereby enhancing treatment effectiveness [12, 13]. This transition is triggered by caspase-3 cleavage of GSDME and regulated by the ASK1/JNK/P38 cascade [14, 15]. AKT, frequently hyperactivated in cancers like ovarian cancer, inhibits this pathway by phosphorylating ASK1 [16–18]. Thus, suppressing AKT may amplify chemo-induced pyroptosis and sensitize tumors.

While inhibiting AKT enhances ovarian cancer cell sensitivity to cisplatin in vitro and in vivo [19, 20]. However, clinical trials targeting AKT directly have not yielded expected outcomes due to tumor heterogeneity, low specificity of AKT inhibitors, and significant side effects [21–28]. This highlights the significance of identifying novel specific targets for AKT inhibition and developing drugs that specifically inhibit AKT activity.

To pinpoint therapeutic targets in cisplatin-resistant ovarian tumors, we employed CRISPR-Cas9 technology screening. Focusing on druggable targets, we primarily investigated secretory proteins. Our study uncovered a novel AKT/mTORC2 binding protein CRLF1 that enhances the interaction between AKT and SIN1, an essential mTORC2 component. This interaction promotes AKT activation and inhibits pyroptosis independent of its secretion, thereby conferring cisplatin resistance to ovarian cancer cells. Interestingly, the overexpression of binding-defective CRLF1 variants effectively increased pyroptosis in a tumor-specific manner. These findings highlight the addiction of ovarian cancer cells to CRLF1 upregulation for inhibiting cisplatin-induced cell death by AKT. The binding-defective CRLF1 variants could be potential tumor-specific polypeptide drugs to enhance chemotherapy effectiveness as adjuvant therapy in ovarian cancer.

## Results

### CRLF1 strengthens chemoresistance in ovarian cancer

**Fig. 1:**
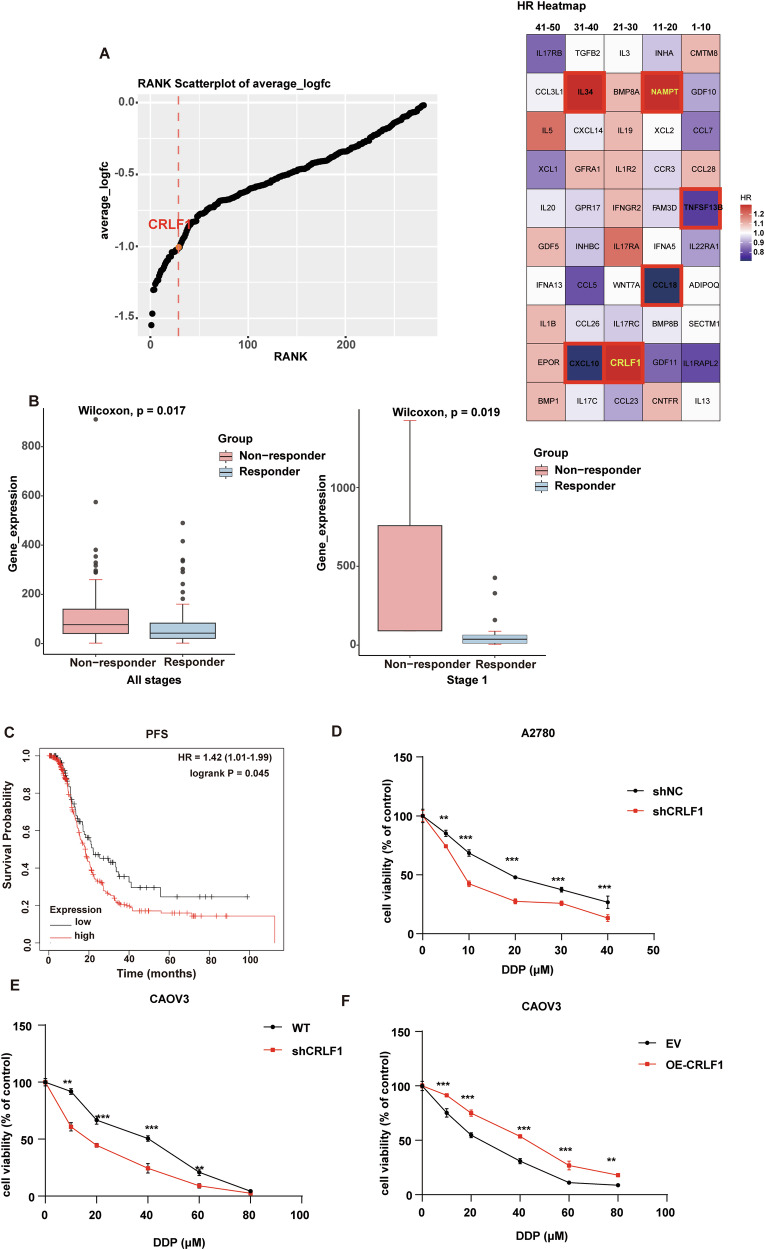
CRLF1 strengthens chemoresistance in ovarian cancer. **A** The candidate genes encoding cytokines were ranked (left) using the STARS algorithm (https://portals.broadinstitute.org/gpp/public/software/stars) based on CRISPR-Cas9 screening data by Elizabeth H. Stover et al. The overall survival analysis of the top 50 cytokine-encoding genes (right) was performed using the GEPIA2 website. **B** Comparatively analyzed the CRLF1 mRNA expression between chemotherapy responder and non-responder in ovarian cancer in ovarian cancer (left) and in stage I tumors (right) using the Kaplan–Meier Plotter website. **C** High CRLF1 level has shorter progression-free survival (PFS) by online Kaplan–Meier analysis. *P < 0.05. Knockdown of CRLF1 reduced cell proliferation in A2780 cells (**D**) and in CaoV3 cells (**E**) and overexpression of CRLF1 promoted cell proliferation in CaoV3 cells (**F**). Cells were treated with an indicated dose of DDP, and cell viability was measured by CCK8 assay at the indicated time points. For graphs, error bars represent mean ± s.e.m. *P* values were determined by unpaired t test unless otherwise indicated, *p < 0.05, ***p < 0.001.

To identify targets involved in chemoresistance in ovarian cancer cells, CRISPR-Cas9 screening data from the ovarian cancer cell line OVSAHO treated with cisplatin (DDP) were analyzed [29]. We screened out cytokines that increased DDP responses after knockout, as they were more druggable. We ranked the negative hits and summarized them in Data S1. Then we analyzed the relationship of those cytokines with prognosis by TCGA data. Among the top 30 cytokines, NAMPT and CRLF1 were positively correlated with hazard risk (poor prognosis) (Fig. 1A). NAMPT has been extensively studied in OC [30, 31]. Previous studies have demonstrated CRLF1 involves in cancer cells proliferation, neuronal development, and immune regulation [32–36]. However, its potential role in regulating programmed cell death pathways and impacting chemoresistance was unknown. To address this, we further investigated CRLF1 function. Analysis of mRNA expression levels demonstrated that CRLF1 exhibited significantly higher expression in chemotherapy non-responders compared to responders, particularly in stage I tumors, as determined through using the KM plotter online analysis (http://kmplot.com/analysis/) (Fig. 1B).

Subsequently, we performed a comprehensive analysis of the progression-free survival (PFS) in a cohort of 340 ovarian cancer patients who underwent debulking surgery followed by chemotherapy using Kaplan–Meier analysis. The results showed that high expression levels of CRLF1 were significantly associated with shorter PFS, suggesting a potential role of CRLF1 in conferring chemoresistance to ovarian cancer (Fig. 1C). Consistent with the above findings, silencing CRLF1 enhanced sensitivity to DDP-induced cytotoxicity in both the DPP-sensitive A2780 and DDP-resistant CaoV3 cell lines (Fig. 1D, E). Moreover, CRLF1 overexpression increased chemoresistance in CaoV3 cells (Fig. 1F). Collectively, these data indicate that high CRLF1 levels confer resistance to DDP in ovarian cancer cells.

### CRLF1 promotes proliferation and invasion in ovarian cancer

**Fig. 2:**
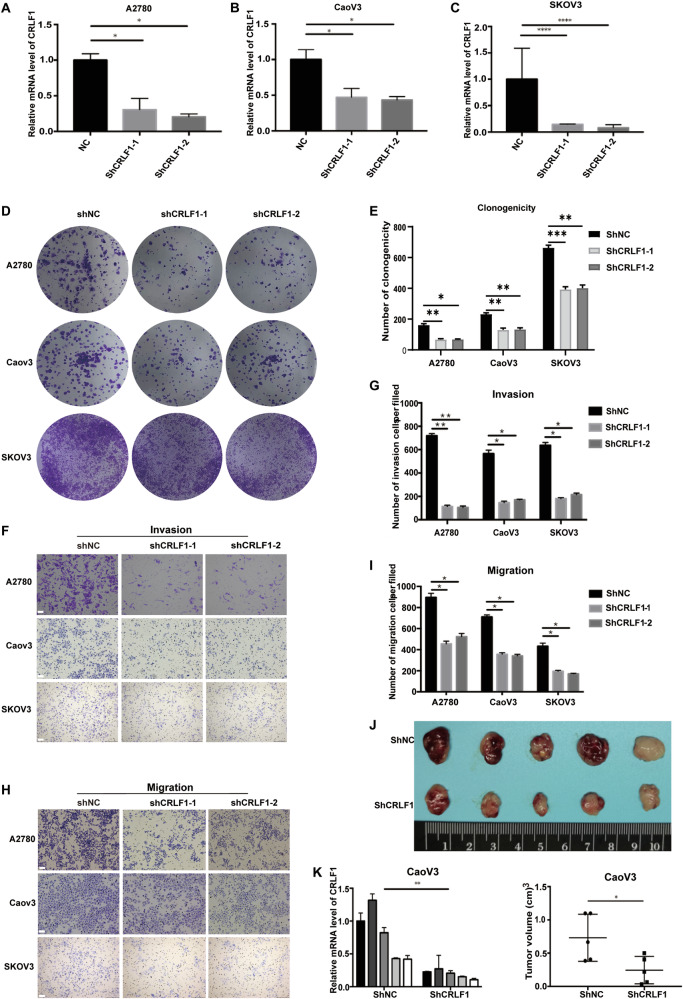
CRLF1 promotes proliferation and invasion in ovarian cancer. Knockdown of CRLF1 in A2780 cells (**A**), CaoV3 cells (**B**), and SKOV3 cells (**C**). The mRNA levels were evaluated 7d after cells were transfected by lentivirus containing the indicated shRNA. **D** Representative images of fixed and stained A2780, CaoV3, and SKOV3 cells were obtained using crystal violet staining to visualize colonies. **E** Colony numbers in (**D**) were counted. Representative images of the indicated cell lines were captured using transwell assays to assess their invasion (**F**) and migration (**H**) capabilities. Scale bar = 20 μm. Quantification of the number of invaded cells (**G**) and migrated cells (**I**). **J** 1.5 × 10^6^ CaoV3 shNC cells and CaoV3 shCRLF1 cells were subcutaneously injection into null mice. Tumors were excised and photographed three weeks after injection. **K** Tumor weight in (**J**) was measured. For graphs, error bars represent mean ± s.e.m. *P* values were determined by one-way ANOVA [(**A**), (**B**), (**C**), (**E**), (**J**), and (**I**)] or unpaired *t* test (**K**), *p < 0.05, ***p < 0.001.

CRLF1 has been implicated in metastasis and proliferation of papillary thyroid carcinoma, [35, 36], but its role in ovarian cancer has not been elucidated. This prompted us to initially investigate the functions of CRLF1 in ovarian cancer progression. Analysis of TCGA database and immunohistochemical staining results revealed that both mRNA and protein expression levels of CRLF1 were upregulated in ovarian cancer (Fig. S1A–C), with further increases observed following DDP treatment (Figs. S1D, 1E). Moreover, higher CRLF1 expression was associated with poorer survival outcomes in ovarian cancer patients (Fig. S1F). Notably, silencing CRLF1 curtailed proliferation, migration, and invasion in ovarian cancer cell lines, including A2780, CaoV3, and SKOV3 (Fig. 2A–H). In addition, CRLF1 silencing suppressed tumor growth in xenograft models (Fig. 2J, K). In brief, CRLF1 functions as an oncogene driving the ovarian cancer cell proliferation and invasion.

### CRLF1 suppresses cisplatin-induced pyproptosis independently of its extracellular secretion

To understand how CRLF1 confers chemoresistance in ovarian cancer cells, we investigated its involvement in the GP130 signaling pathway. CRLF1, a secreted cytokine, forms a heterodimer with CLCF1, activating the ERK/MAPK cascade through the CNTFR/GP130/JAK receptor complex, promoting cell proliferation [33, 37, 38]. However, the role of this pathway in chemoresistance remains unclear. We conducted knockdown experiments of CLCF1 and CNTFR in A2780 and CaoV3 cell lines, confirming their efficiency through Real-time fluorescence quantitative PCR (qRT-PCR) (Figs. S1A, 2B). Our results showed that CLCF1 or CNTFR knockdown did not affect DDP-induced cytotoxicity in ovarian cancer cells (Figs. S2C, 2D). Additionally, inhibiting ERK increased DDP IC50 by ~10% in both control and CRLF1 knockdown cells (Fig. S2E–H). These findings indicate that the known functions of CRLF1 do not mediate chemoresistance.

**Fig. 3:**
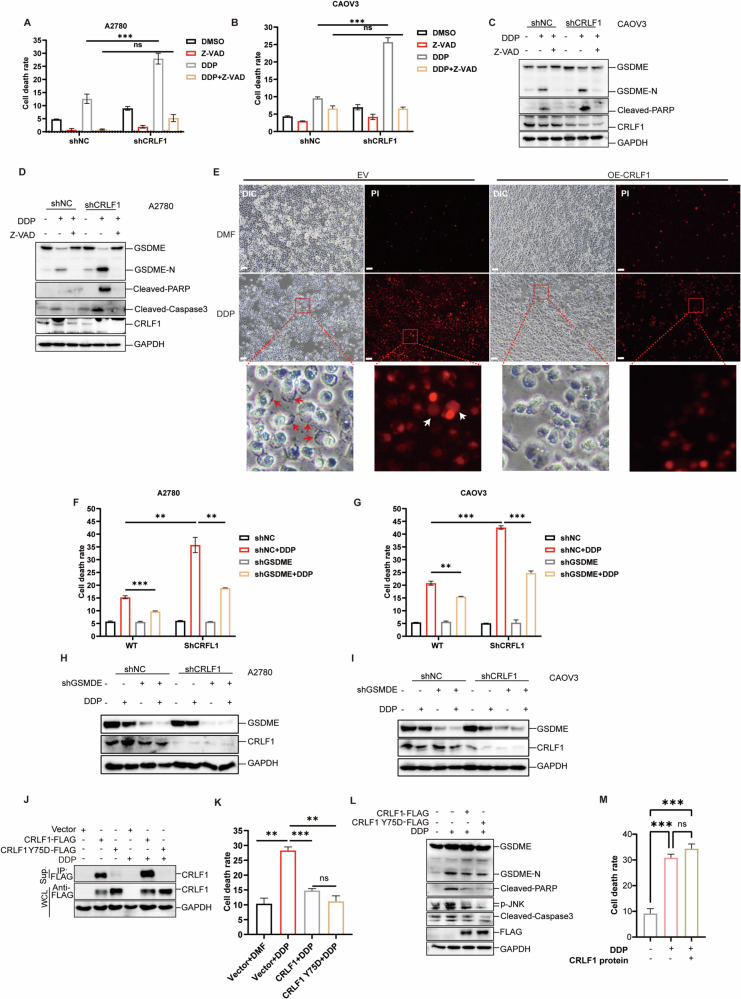
CRLF1 suppresses cisplatin-induced pyproptosis independently of its extracellular secretion. Knockdown of CRLF1 dampened DDP-induced cell death. The indicated cell lines were treated with IC50 dose of DDP for 18 h. Cell death was assessed using lactate dehydrogenase (LDH) assay (**A**, **B**) and western blotting (**C**, **D**) for A2780 cells and CaoV3 cells, respectively. **E** Microscopic images of CaoV3 cells transfected with expression constructs for CRLF1 or empty vector, followed by DDP or DMF treatment for 24 h. Arrows indicate ballooned cell membrane characteristic of pyroptotic cells. Scale bar, 100 μm. Knockdown of GSDME resulted in a significant reduction in cell death proportion in shCRLF1 cells. Cell death was evaluated by LDH release (**F**, **G**) and GSDME expression levels were assessed by western blotting (**H**, **I**) for A2780 and CaoV3 cells, respectively. **J** Immunoprecipitation was employed to evaluate the secretion potential of supernatant CRLF1 Y75D in the presence or absence of DDP. Overexpression of CRLF1 Y75D mutant inhibited DDP-induced cell death. Cell death was assessed by LDH assay (**K**) and western blotting (**L**). **M** 20 nM of purified CRLF1 protein was introduced into the cell culture medium 24 h prior to DDP treatment. Cell death was assessed using the LDH assay. For all graphs, error bars represent mean ± s.e.m. *P* values were determined by one-way ANOVA unless otherwise indicated, *p < 0.05, ***p < 0.001.

CRISPR-Cas9 screening suggested that CRLF1 more likely directly inhibits cell death to enhance chemoresistance in ovarian cancer. Comparing CRLF1 knockdown and control cells treated with DDP, we observed that silencing CRLF1 accelerated apoptosis and pyroptosis (Fig. 3A–D). Assessing the morphological characteristics of cell death induced by DDP, we found that DDP treatment led to pronounced cell death with pyroptotic features, which were effectively suppressed by CRLF1 overexpression (Fig. 3E). Furthermore, knocking down GSDME, a pyroptosis executor, in both CRLF1 knockdown and control cells followed by DDP treatment showed a more significant reduction in cell death proportion in CRLF1 knockdown cells compared to control cells (Fig. 3F–I). These results demonstrate that CRLF1 primarily suppresses pyroptosis to reduce cell death.

As a secretory protein, CRLF1 interacts with cell membrane receptors to activate downstream pathways. We investigated whether extracellular secretion is essential for CRLF1 through the modulation of unidentified receptor-mediated signaling pathways. Previous studies have identified a CRLF1 mutant with a tyrosine (Y) to serine (D) substitution at position 75, which impairs its secretion [32, 37]. Confirming these findings, our study showed that despite DDP treatment, no CRLF1 Y75D protein in the supernatant was detected, possibly due to CRLF1 Y75D inhibiting pyroptosis, preventing its leakage into the supernatant. (Fig. 3J–L). The introduction of CRLF1 protein into the cell culture medium had no discernible impact on DDP-induced cell death (Fig. 3M). In summary, our findings demonstrate that CRLF1 enhances chemoresistance by directly inhibiting pyroptosis, independent of its extracellular secretion.

### CRLF1 attenuates pyroptosis via restraining the ASK1/JNK/Caspase3/GSDME cascade

**Fig. 4:**
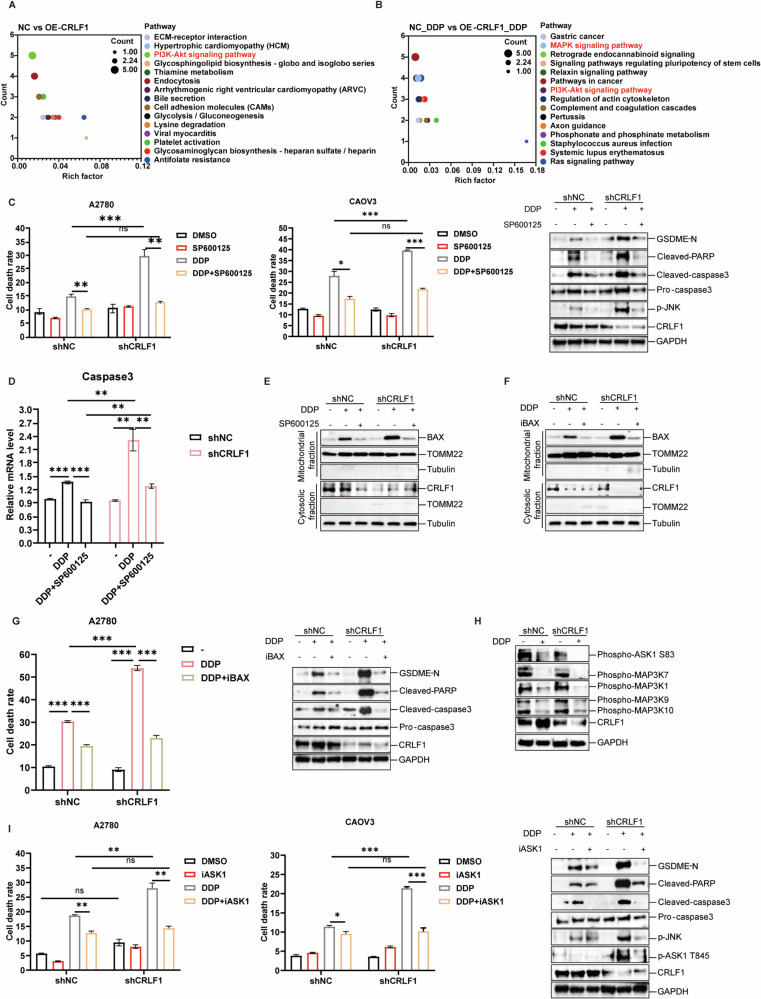
CRLF1 Attenuates pyroptosis via inhibiting the ASK1/JNK/Caspase3/GSDME signaling pathway. KEGG pathway analysis demonstrated that the MAPK signaling pathway was exclusively enriched in the DDP treatment group (**B)** compared to the absence of DDP (**A**). The top 15 enriched pathways were displayed, with PI3K and MAPK highlighted in red. The size of the circles represents the number of DEGs, and the color indicates the enriched pathways. **C** The indicated cell lines were treated with 10 μM SP600125, a JNK inhibitor, followed by DDP treatment for 18 h. Cell death in A2780 cells and CaoV3 cells was assessed using LDH assay and western blotting. **D** The caspase-3 mRNA levels were assessed in the indicated cells treated with DPP in the presence or absence of SP600125. Mitochondria from the indicated cells treated with DPP in the presence or absence of SP600125 (**E**) or iBAX (**F**) were isolated, and mitochondrial BAX was subsequently analyzed by western blotting. **G** The indicated cell lines were treated with 50 μM iBAX, a BAX inhibitor, followed by DDP treatment for 18 h. Cell death was assessed using LDH assay and western blotting. **H** The phosphorylation levels of MAP3Ks upstream of JNK1 were quantified in designated cell models upon DDP exposure and in its absence. **I** Cell death was assessed after treating the specified cell lines with 5 μM ASK1 inhibitor, followed by 18-h DDP treatment. LDH assay and western blotting were utilized for evaluation. For graphs, error bars represent mean ± s.e.m. *P* values were determined by one-way ANOVA unless otherwise indicated, *p < 0.05, ***p < 0.001.

We discovered that CRLF1 confers chemoresistance by inhibiting pyroptosis instead of enhancing proliferation. This led us to explore whether CRLF1 functions in a context-dependent manner. We conducted RNA sequencing analysis compared A2780 cells overexpressing CRLF1 with controls, with or without DDP. The results revealed significant differences in the differentially expressed genes regulated by CRLF1 under DDP or non-DDP conditions (Figs. S3A, 3B). Cluster analysis revealed that CRLF1 mainly upregulated genes in cluster 3, while gene expression in clusters 1 and 2 did not exhibit a consistent pattern across groups. These upregulated genes were predominantly related to neuron protection and development in the non-DDP condition (Fig. S3C). However, in the DDP condition, CRLF1-regulated genes were closely linked to limb development and endoplasmic reticulum stress response (Fig. S3D). This was corroborated by GO and KEGG pathway enrichment analyses (Figs. S3E, 3F, and 4A, B), which indicated that CRLF1’s target genes and pathways might change depending on the context. Moreover, our findings revealed that among the cell death-regulated pathways, the MAPK pathway was uniquely enriched in the DDP-treated group (Fig. 4A, B), implying a possible key role of the CRLF1-mediated MAPK pathway in pyroptosis inhibition.

Chemotherapy drugs frequently activate downstream MAPK proteins JNK and P38 mediated by ASK1 [38–40]. Activated JNK facilitates transcriptional upregulation and activation of caspase-3, leading to apoptosis and cleaving GSDME for pyroptosis [12, 41–43]. The JNK1 blockade, unlike P38 (Fig. S4A–D), countermands the CRLF1-mediated repressing effect on DDP-induced pyroptosis (Fig. 4C). CRLF1 knockdown facilitated caspase-3 transcription and BAX mitochondrial translocation via phosphorylated JNK1 (Fig. 4D, E). Blocking BAX translocation negated CRLF1’s pyroptotic effects (Fig. 4F, G). Furthermore, CRLF1 silencing augmented ASK1 activity, distinct from other MAP3Ks upstream of JNK1 (Fig. 4H). ASK1 inhibition also mitigated CRLF1’s inhibitory effect on DDP-induced pyroptosis (Fig. 4I). Collectively, these data reveal that CRFL1 attenuates pyropotosis by constraining ASK1/JNK/caspase3/GSDME pathway.

### CRLF1-mediated enhancement of AKT phosphorylation inhibits pyroptosis

To further investigate the mechanisms by which CRLF1 suppresses ASK1 activity, we examined the role of CRLF1 in the regulation of ROS production. Drug-induced ROS oxidizes thioredoxin (Trx), a redox-responsive protein, leading to its dissociation from AKS1 and subsequent activation of ASK1 through autophosphorylation [44–46]. Surprisedly, knockdown of CRLF1 did not affect ROS production induced by DDP (Fig. S5A–D).

**Fig. 5:**
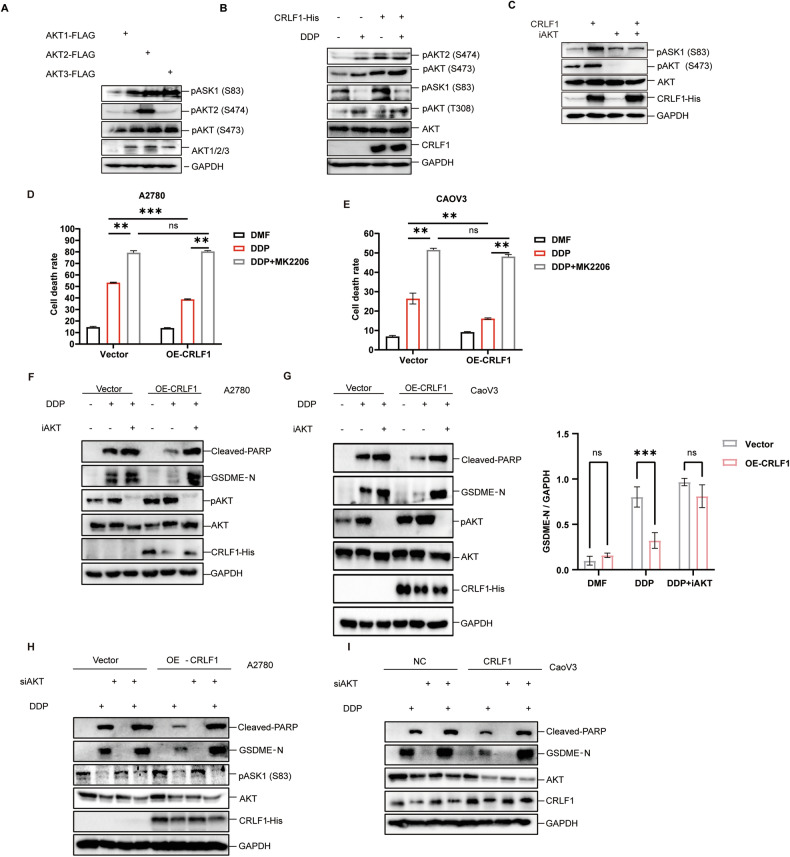
CRLF1 reduces pyroptosis by augmenting AKT phosphorylation level. **A** A2780 cells were subjected to overexpression of AKT1, AKT2, and AKT3. Phosphorylation levels of ASK1 at Ser 83, AKT2 at Ser 474, and AKT1 at Ser 473 were assessed using specific antibodies. The antibody targeting phosphorylated AKT1 at Ser 473 also detects phosphorylated AKT2 and AKT3 at their corresponding residues. **B** Overexpression of CRLF1 resulted in increased phosphorylation levels of ASK1 at Ser 83 and AKT at Ser 473, while AKT phosphorylation at Thr 308 remained largely unaffected. A2780 cells were transfected with either a plasmid vector or a plasmid overexpressing CRLF1, followed by the addition of DDP to the medium 24 h after transfection. **C** Cells were transfected with either an empty vector or a CRLF-expressing vector, and then treated with an AKT inhibitor for 24 h. (**D**–**G**) A2780 and CaoV3 cells were treated with 10 μM MK2206, a pan-AKT inhibitor, followed by an 18-h DDP treatment 30 min after adding the AKT inhibitor. Cell death was assessed using LDH assays for A2780 cells (**D**) and CaoV3 cells (**E**). Protein analysis was performed using western blotting in A2780 cells (**F**) and CaoV3 cells (Fig. **G,** left), while quantification of GSDME-N was conducted through Image J (Fig. **G,** right). For graphs, error bars represent mean ± s.e.m. *P* values were determined by two-way ANOVA, *p < 0.05, ***p < 0.001. A2780 (**H**) and CaoV3 (**I**) cells were simultaneously silenced for AKT1/2/3, followed by a 24-h treatment with the IC50 dose of DDP. Cleaved-PARP and cleaved-GSDME, indicators of apoptosis and pyroptosis respectively, were detected using western blotting to assess the cellular response.

AKT2 has been reported to inhibit ASK1 by phosphorylating it at Ser 83 [16]. We first explored the inhibitory role of all AKT isoforms on ASK1, considering their structural and functional similarities [47, 48]. By overexpressing AKT1, AKT2, and AKT3, we observed that all isoforms were capable of inhibiting ASK1 activity through increasing the phosphorylation of ASK1 at Ser 83 (Fig. 5A). Subsequently, we explored whether CRLF1 suppresses ASK1 activity by enhancing AKT phosphorylation. As expected, overexpression of CRLF1 enhanced the phosphorylation of both ASK1 at Ser 83 and AKT at Ser 473, as detected by an anti-phospho AKT1 (Ser473) antibody that also recognizes phosphorylated AKT2 and AKT3 at corresponding residues (Fig. 5B). However, phosphorylation at Thr 308 was almost not affected, which aligns with previous findings emphasizing the importance of AKT phosphorylation at Ser 473 for cell survival [48–50]. AKT inhibition abrogates CRLF1-enhanced ASK1 S83 phosphorylation (Fig. 5C). Collectively, these data suggest CRLF1 promotes ASK1 S83 phosphorylation via AKT phosphorylation enhancement.

To confirm the dependence of CRLF1-mediated pyroptosis inhibition on AKT phosphorylation, we either knocked down all AKT isoforms or inhibited their activity simultaneously, followed by DDP treatment. Remarkably, the knockdown (Fig. 5H, I) or inhibition of AKT isoforms (Fig. 5D–G) reversed CRLF1-mediated pyroptosis inhibition. Blockade of other AKT downstream targets did not reverse CRLF1’s restraint on pyroptosis (Fig. S6A–D). In summary, our data reveal that CRLF1 enhances AKT phosphorylation at Ser 473, thereby mitigating ASK1/JNK-driven pyroptosis.

### CRLF1 enhances chemoresistance and increases susceptibility to AKT inhibitors in mice xenografts

**Fig. 6:**
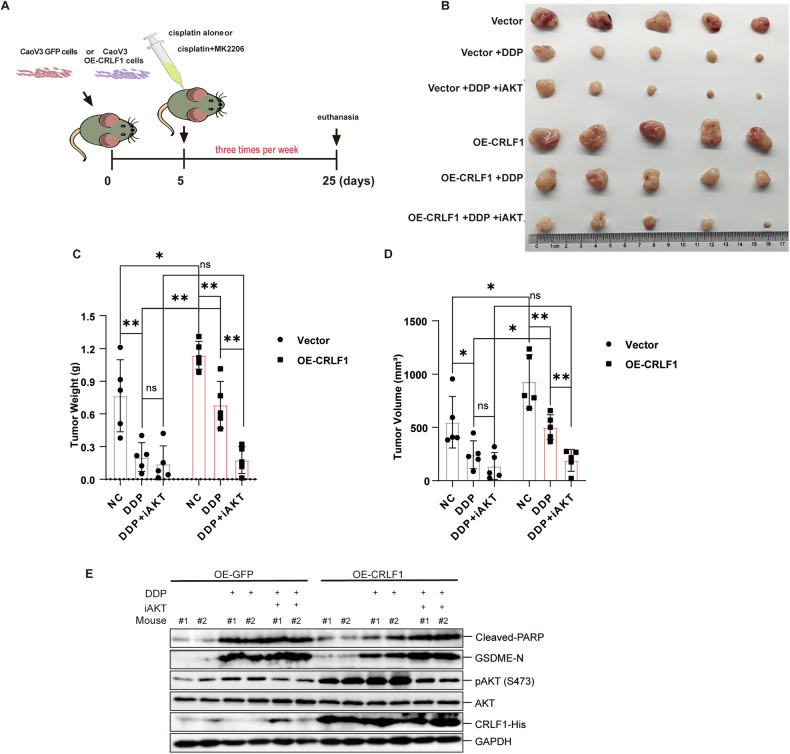
CRLF1 enhances chemoresistance and sensitivity to AKT inhibitor in mouse xenografts. **A** Workflow schematic of drug treatments in mice. **B** CaoV3 ovarian cancer xenografts were treated with DDP alone at doses of 4 mg/kg or in combination with MK2206 at 120 mg/kg, administered three times per week. Tumors were excised and photographed 25 days after subcutaneous injection. (*N* = 5). Tumor weight (**C**) and tumor volume (**D**) were calculated. The formula for calculating tumor volume is length x width^2^/2. **E** The indicated protein levels in tumors were analyzed by western blotting. Error bars in the graphs represent mean ± s.d. Statistical significance indicated as *p < 0.05, ***p < 0.001 by one-way ANOVA.

To determine whether CRLF1 could augment chemoresistance by enhancing AKT phosphorylation in vivo, mice were subcutaneously injected with either 1.5 × 10^6^ control cells or cells overexpressing CRLF1(CaoV3). The mice were then treated with or without DDP, and further received an intraperitoneal injection of an AKT inhibitor or control CMC-Na (Fig. 6A). Tumor growth was significantly inhibited in control mice treated with DDP versus controls (Fig. 6B). However, CRLF1-overexpressing tumors were larger and more resistant to DDP. In control mice, tumor weight and volume were reduced in mice treated with DDP + MK2206 (an AKT inhibitor) versus DDP alone, with no significant difference (Fig. 6C, D). In contrast, CRLF1-overexpressing tumors showed a significant reduction in weight and volume with DDP + MK2206 versus DDP alone. There was no difference between control and CRLF1 groups when treated with DDP + MK2206 (Fig. 6C, D). Western blot analysis demonstrated increased p-AKT levels and decreased cleaved-PARP and cleaved-GSDME levels in DDP-treated tumors overexpressing CRLF1 (Fig. 6E). The AKT inhibitor effectively reversed effects mediated by CRLF1, suggesting its role modulating pyroptosis via AKT phosphorylation in vivo. In summary, CRLF1 enhances chemoresistance by inhibiting pyroptosis and increasing AKT inhibitor susceptibility in ovarian cancer.

### CRFL1 augments AKT phosphorylation by promoting interaction between AKT and SIN1

**Fig. 7:**
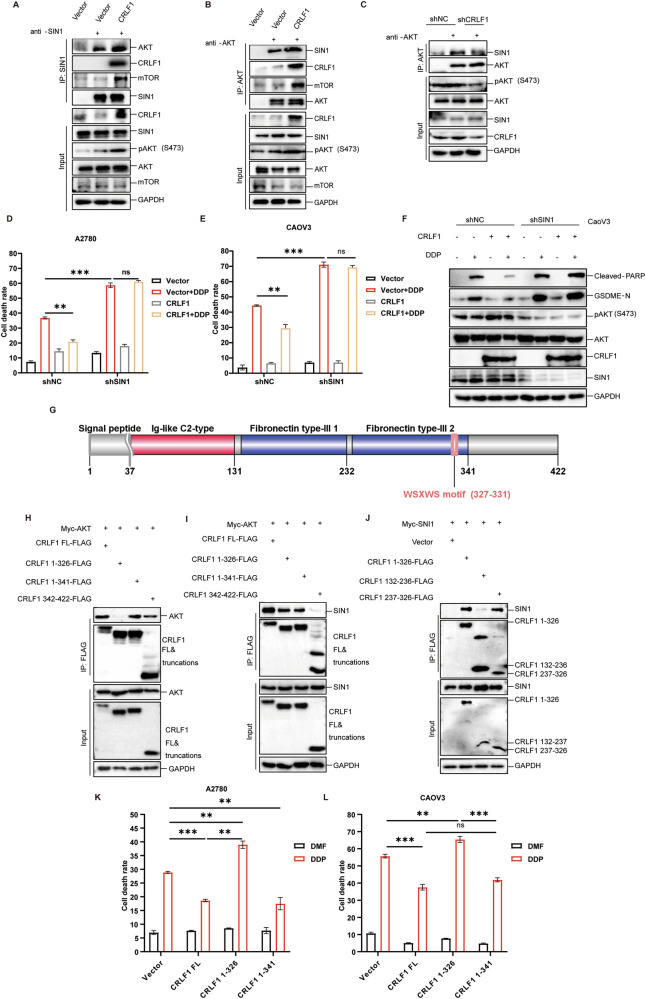
CRLF1 enhances AKT phosphorylation through facilitating the interaction between AKT and SIN1. Overexpression of CRLF1 in 293T cells, followed by co-immunoprecipitation using antibodies against AKT (**A**) or SIN1 (**B**) to assess the interaction between AKT and SIN1 in the presence or absence of CRLF1. **C** CRLF1 knockdown was performed to evaluate the strength of the interaction between AKT and SIN1 using a co-immunoprecipitation assay. (**D**–**F**) To assess the impact of SIN1 on CRLF1-mediated pyroptosis, we performed ectopic expression of CRLF1 in cells with normal SIN1 expression and cells with SIN1 knockdown. Cell death was measured using LDH assay in A2780 (**D**) and CaoV3 cells (**E**). Additionally, western blotting was employed to analyze cleaved-PARP and cleaved-GSDME (**F**). **G** A schematic diagram illustrating the domain, motif, and region of CRLF1. To investigate the interaction regions of CRLF1 with AKT and SIN1, specific CRLF1 variants were ectopically expressed with either AKT or SIN1. Co-immunoprecipitation assays revealed that the CRLF1 aa 327-422 region interacted with AKT (**H**), while the CRLF1 aa 237-326 region interacted with SIN1 (**I**, **J**). A2780 (**K**) and CaoV3 cells (**L**) were transfected with wild-type (WT) CRLF1 and its variants. The AKT-binding-defective CRLF1 variant (CRLF1 aa 1-326) reversed CRLF1-mediated pyroptosis. Cell death was assessed using LDH assay. For graphs, error bars represent mean ± s.e.m. *P* values were determined by one-way ANOVA, *p < 0.05, ***p < 0.001.

The mTORC2 complex, facilitated by SIN1, forms an unstable complex with AKT, leading to AKT phosphorylation at Ser 473 [48]. Given that CRLF1 lacks enzymatic activity, we hypothesized that CRLF1 acts as an adaptor to strengthen the interaction between AKT and SIN1, promoting AKT phosphorylation. Co-immunoprecipitation assays confirmed that CRLF1 interacts with AKT and SIN1, enhancing their interaction (Fig. 7A, B). CRLF1 overexpression also increased the co-precipitation of mTOR with AKT and SIN1 (Fig. 7A, B). Importantly, CRLF1 failed to enhance AKT phosphorylation or inhibit DDP-induced pyroptosis when SIN1 was silenced (Fig. 7D–F). These results provide evidence that CRLF1 enhances AKT phosphorylation by strengthening the AKT-SIN1 interaction.

To identify the specific domains of CRLF1 that mediate the interaction with AKT and SIN1, we used AlphaFold prediction to analyze the structure of CRLF1. CRLF1 consists of an N-terminal signal peptide, Ig-like domain, fibronectin repeats with a conserved WSXWS motif, and a disordered C-terminal region (Fig. 7G). The WSXWS motif (aa 327-331) of CRLF1 appearing to play a crucial role in protein binding [51]. To evaluate the involvement of the WSXWS motif in the interaction with AKT and SIN1, we generated three CRLF1 variants: CRLF1 aa 1-326 (lacking the WSXWS motif), CRLF1 aa 1-341 (containing the WSXWS motif), and CRLF1 aa 342-422 variants (disordered C-terminal region). Co-IP assays with CRLF1 variants revealed that the WSXWS motif and the disordered C-terminal region (CRLF1 aa 327-422) are required for the interaction with AKT, while the CRLF1 aa 1-326 region specifically interacts with SIN1 (Fig. 7H, I). To further narrow down the region of CRLF1 that binds to SIN1, we divided the CRLF1 aa 1-326 fragment into three variants: CRLF1 aa 1-131, CRLF1 aa 132-236, and CRLF1 aa 237-326. Unexpectedly, intracellular expression of the CRLF1 aa 1-131 truncation was not detected (data not shown), suggesting that it might be secreted extracellularly. Co-IP assays revealed that the CRLF1 aa 237-326 variant, but not the CRLF1 aa 132-236 variant, interacted with SIN1 (Fig. 7J). These results demonstrate that the CRLF1 aa 327-422 region mediates interaction with AKT, while the CRLF1 aa 237-326 region binds to SIN1.

To confirm the functional significance of the simultaneous interaction of CRLF1 with both AKT and SIN1, we overexpressed full-length CRLF1 (FL), CRLF1 aa 1-326, and CRLF1 aa 1-341 variants and assessed their effects on DDP-induced pyroptosis. As expected, overexpression of CRLF1 FL and CRLF1 aa 1-341 variant effectively inhibited DDP-induced pyroptosis (Fig. 7K, L). In contrast, overexpression of the aa 1-326 variant, lacking the AKT interaction region, not only failed to inhibit pyroptosis but unexpectedly enhanced its occurrence (Fig. 7K, L). These results highlight the critical role of CRLF1 as a bridge between AKT and SIN1, which is necessary for its anti-pyroptotic function.

### Overexpression of binding-defective CRLF1 variants enhance DDP-induced pyroptosis specifically in ovarian cancer cells

**Fig. 8:**
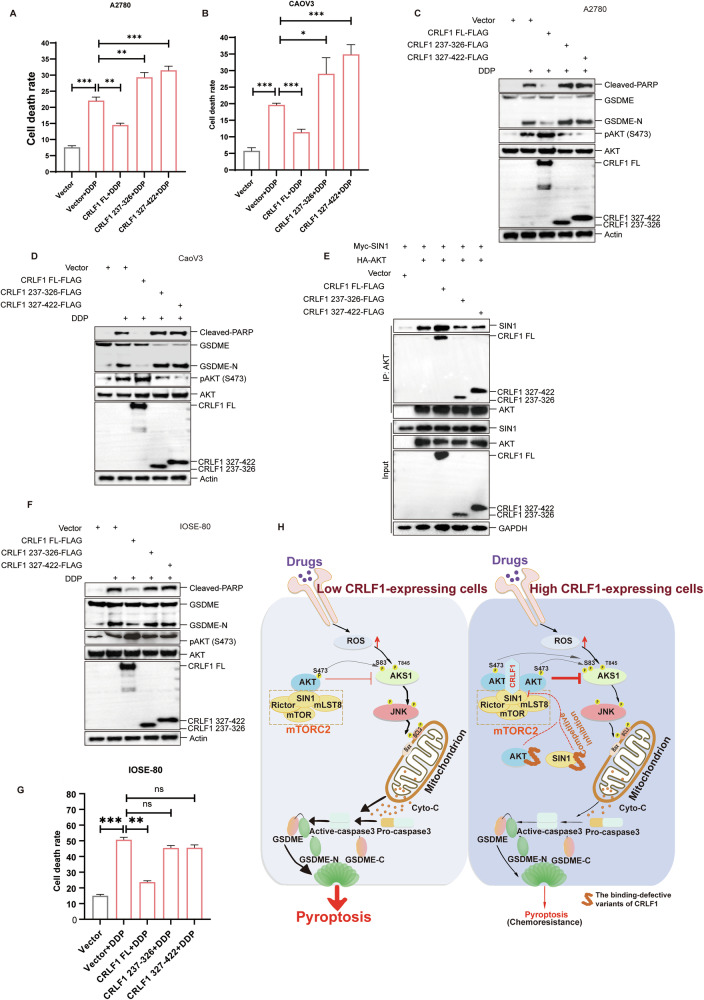
Overexpression of binding-defective CRLF1 variants promote DDP-induced cell death in a tumor-specific way. (**A**–**D**) Overexpression of AKT-binding defective and SIN1-binding defective CRLF1 variants attenuated DDP-induced pyroptosis. LDH assay was used to measure cell death in A2780 (**A**) and CaoV3 cells (**B**). Cleaved-GSDME was detected to analyze pyroptosis in A2780 (**C**) and CaoV3 cells (**D**). **E** Co-immunoprecipitation assay was performed to evaluate the effects of binding-defective CRLF1 variants on the interaction between AKT and SIN1, as well as AKT phosphorylation levels. LDH assay (**F**) and western blotting (**G**) were employed to assess cell death induced by overexpression of binding-defective CRLF1 variants in IOSE-80 cells. **H** Schematic representation of CRLF1 sensitizes ovarian cancer cells to chemotherapy-induced pyroptosis. CRLF1 acts as an adaptor, enhancing the AKT-SIN1 interaction, promoting AKT phosphorylation, and subsequent pyroptosis induction upon chemotherapy drug exposure. Overexpression of binding-defective CRLF1 variants acts as competitive inhibitors, disrupting the AKT-SIN1 interaction, thereby suppressing AKT phosphorylation and preventing pyroptosis induced by drugs. Error bars represent mean ± s.e.m. Statistical significance denoted as *p < 0.05, ***p < 0.001 by one-way ANOVA.

We observed that a CRLF1 variant (CRLF1 aa 1-326) disrupting its binding to AKT increased DDP-induced pyroptosis instead of reducing it. This prompted us to investigate if another CRLF1 variant impairing its binding to SIN1 had a similar effect. We overexpressed full-length CRLF1 (CRLF1 FL), a truncated AKT-binding defective variant (CRLF1 aa 237-326), and an SIN1-binding defective variant (CRLF1 aa 327-422) to assess their impact on cell death. Our results showed that CRLF1 FL overexpression inhibited DDP-induced cell death, while the binding-defective variants (CRLF1 aa 237-326 and CRLF1 aa 327-422) enhanced it (Fig. 8A, B). Western blot analysis revealed increased cleavage of GSDME and PARP in cells overexpressing these binding-defective variants (Fig. 8C, D). Interestingly, the overexpression of the variants also reduced AKT phosphorylation levels (Fig. 8C, D).

Based on these findings, we hypothesized that the binding-defective CRLF1 variants act as competitive inhibitors weakening the interaction between SIN1 and AKT. This inhibition suppresses AKT phosphorylation and subsequent pyroptosis. To validate this hypothesis, we conducted a Co-IP assay, confirming that overexpression of the binding-defective CRLF1 variants diminished this interaction (Fig. 8E). In conclusion, overexpression of binding-defective CRLF1 variants sensitizes ovarian cancer cells to DDP-induced pyroptosis by competitively inhibiting the AKT-SIN1 interaction and reducing AKT phosphorylation.

To explore whether these variants exert their functions in a tumor-specific or CRLF1 expression level-dependent manner, we compared CRLF1 expression levels in IOSE-80 (a normal ovarian epithelial cell line) with A2780 and CaoV3 cells. Results showed that IOSE-80 cells expressed lower levels of CRLF1 despite higher levels of SIN1 and AKT expression compared to A2780 and CaoV3 cells (Fig. S7A). Interestingly, overexpression of binding-defective CRLF1 variants inhibited the proliferation of A2780 and CaoV3 cells but had no effect on IOSE-80 cell proliferation (Fig. S7B–E). As expected, overexpression of binding-defective CRLF1 variants did not enhance DDP-induced cell death in IOSE-80 cells compared to the control group (Fig. 8F, G). These findings suggest that binding-defective CRLF1 variants have the potential to serve as tumor-specific polypeptide drugs, enhancing chemotherapy effectiveness as adjuvant therapy in ovarian cancer (Fig. 8H).

## Discussion

Although various strategies have been employed for the treatment of ovarian cancer, such as immunotherapy and hormone therapy, the prevailing standard approach entails debulking surgery combined with platinum-based chemotherapy, particularly cisplatin [52–55]. However, the efficacy of chemotherapy is hampered by the propensity of ovarian cancer cells to develop resistance to drug-induced cell death [6]. Efforts focused on increasing the susceptibility of cancer cells to programmed cell death pathways, particularly pyroptosis, offer a promising avenue for overcoming chemoresistance [7, 8]. Our investigation unveils CRLF1, a secreted protein, as a novel regulator influencing pyroptosis and cisplatin resistance in ovarian cancer independent on its secretion.

The secretory protein CRLF1 complexes with cardiotrophin-like cytokine (CLCF1), P28, or soluble ciliary neurotrophic factor receptor, leading to subsequent interactions with cell membrane receptors, thereby activating the JAK/ERK and AKT signaling pathways [56, 57]. CRLF1 plays a crucial role during neuronal development and also participates in immune regulation, and proliferation and metastasis in PTC [32–36]. However, its involvement in programmed cell death regulation and the precise mechanisms orchestrating CRLF1’s activation of the PI3K/AKT pathways in ovarian cancer remain obscure.

Our research shows high CRLF1 expression in ovarian cancer correlates with poor patient survival and chemoresistance. Mechanistically, CRLF1 impedes pyroptosis via the ASK1/JNK/caspase-3/GSDME pathway. RNA sequencing uncovered context-dependent regulatory mechanisms. Inhibiting ASK1 suppresses downstream JNK and P38 activation [40]. Notably, our results indicate that specifically inhibiting JNK, but not P38, reversed CRLF1-mediated pyroptosis, implying it differentially regulates these pathways via other mechanisms. It has been reported that inhibiting JNK in certain cases could enhance drug-induced cell death [58–61]. These discrepancies may stem from variations in JNK subtype expression levels, drug concentration, treatment duration, and cellular models employed.

While chemotherapy drugs typically activate ASK1 by inducing ROS production [38, 62], our investigations reveal that CRLF1 does not affect DDP-induced ROS production. Instead, CRLF1 functions as an adaptor protein, strengthening the interaction between AKT and the mTORC2 subunit SIN1. This enhanced binding augments AKT phosphorylation by mTORC2, fortifying AKT’s inhibition of the ASK1/JNK/caspase-3/GSDME cascade. Consequently, CRLF1 stifles pyroptosis by augmenting mTORC2-mediated AKT activation, providing ovarian cancer cells with a survival advantage against cisplatin cytotoxicity.

Directly targeting AKT has encountered clinical challenges due to tumor heterogeneity, genetic alterations, limited specificity, and significant side effects, compromising the efficacy and clinical utility of AKT inhibitors [21, 22, 24, 25, 27, 28]. Our findings suggest CRLF1 may represent a more specific and effective alternative target. We found CRLF1 knockdown or overexpression of a binding-defective CRLF1 variant could effectively block the stabilization of AKT-SIN1 interaction, leading to reduced AKT phosphorylation and increased ASK1 activity. This ultimately restores ovarian cancer cell susceptibility to cisplatin-induced pyroptosis, shed light on the crucial role of CRLF1’s adaptor function in regulating AKT activation and chemoresistance. Targeting CRLF1 might enable AKT inhibition with greater specificity towards cancer cells.

AKT activation induced by CRLF1 provides the treatment strategy for those OC patients with highly expressed CRLF1 by AKT inhibitors, which was already confirmed in vivo assay. Remarkably, binding-defective CRLF1 variants promoted tumor-specific pyroptosis and chemosensitization by impairing the AKT-SIN1 interaction. This selective and specific targeting potentially avoided the side effects caused by AKT inhibitors. These variant proteins could serve as innovative polypeptide drugs to enhance chemotherapy, representing a promising new class of adjunct therapies.

Future studies should directly disrupt the AKT-SIN1 interaction in vivo as an adjuvant therapy to enhance ovarian cancer cell pyroptosis and overcome platinum resistance. We have delineated distinct AKT and SIN1 binding regions of CRLF1, providing a foundation for designing inhibitors or targeting mutants. Furthermore, evaluating the specificity and cytotoxicity of CRLF1 inhibition in normal cells is imperative. If proven safe and efficacious, CRLF1-targeted therapy alongside platinum-based chemotherapy could significantly improve patient outcomes. Additionally, CRISPR screening of other AKT-modulating factors may uncover other synergistic targets. Systems approaches may also define patient subgroups most likely to benefit. Overall, this work elucidates a novel CRLF1-SIN1/AKT-pyroptosis axis that drives chemoresistance in ovarian cancer, firmly establishing CRLF1 as a promising therapeutic target warranting further exploration.

## Methods and materials

### Plasmids

Human CRLF1 cDNA was obtained by PCR, and then C-terminal His-tagged CRLF1 was constructed in the lentiviral expression vector pCDH. The CRLF1 truncations, such as CRLF1 1-131-FLAG, CRLF1 132-326-FLAG, CRLF1 132-341-FLAG and CRLF1 327-422-FLAG, were constructed by subcloning the corresponding CRLF1 complementary DNAs from CRLF1-His into pRK5F vector. The FLAG-AKT1, FLAG-AKT2 and FLAG-AKT3 was a kind gift from Xinhua Feng. ShRNA vectors that knocked down endogenous CRLF1, GSDME, and SIN1 were constructed in pLKO.1 lentiviral vector. The specific target sequences of these genes are listed as follows: shCRLF1#1 (5′-CTTTGGCATCTATGGCTCCAA-3′), shCRLF1#2 (5′-CGATGTACTCACGCTGGATAT-3′), shGSDME#1 (5′-GCATTCATAGACATGCCAGAT-3′), shGSDME#2 (5′-GCATGATGAATGACCTGACTT-3′), shSIN1#1 (5′-AAGAGTCACTCTTTGTTCGAA-3′), and shSIN1#2 (5′-AAGACGCTCAAACACAGCTCA-3′).

### Antibodies and reagents

Antibodies specific for Akt (9272), phospho-Akt (Ser473, 4060S), phospho-Akt (Thr308, 13038S), phospho-Akt2 (Ser474, 8599S), phospho-ASK1 (Thr845, 3765S), phospho-FOXO1, phospho-S6K, S6K, mTOR (2983S), Cleaved Caspase-3 (9661S), and Cleaved-PARP (5625S) were obtained from Cell Signaling Technology. Anti-FLAG-tag (20543-1-AP), anti-mouse (SA0001-7), anti-rabbit (SA0001-9), Phospho-p38 MAPK (Thr180/Tyr182), Phospho-MAP3K1 (Thr1400), Phospho-TAK1 (Thr187), anti- MDM2, anti-caspase3 and anti-SIN1-tag (15463-1-AP) were purchased from Proteintech. Anti-His-tag (AE086), p38 MAPK (A14401), Phospho-HSP27-S82 (AP0041), anti-Phospho-JNK1/2-T183/Y185 + JNK3-T221/Y233 (AP1337), anti-XIAP, anti-FOXO1 and Phospho-ASK1-S83 (AP0059) were purchased from Abclonal. Anti-GSDME (ab215191), anti-c-Myc and anti-c-Myc (phospho T58) were purchased from Abcam. Anti-GAPDH (PTM-5150) was purchased from PTM BIO (Hnagzhou, China). Anti-CRLF1 (NBP1-85606) was purchased from Novus Biologicals. Anti-phospho MAP3K9/10 (Thr312/266) were purchased from JiNingShiYe Ltd. (Shanghai, China). Inhibitors: Z-VAD-FMK, MK-2206, Selonsertib, SP 600125, cisplatin, Bax inhibitor peptide V5, CHIR-99021, MX69, and SB202190 were purchased from Selleck. Rheb inhibitor NR1 and JY-2 were purchased from MedChemExpress. CytoTox 96® Non-Radioactive Cytotoxicity kit (G1780) was purchased from Promega. Q5® Site-Directed Mutagenesis Kit (E0554) and Q5® High-Fidelity DNA Polymerases were purchased from NEW ENGLAND BioLabs (NEB). Ceturegel Matrix LDEV-Free matrix gel (40183ES10) was purchased from YEASEN (Shanghai, China). Proteinase Cocktail (B14001) was purchased from BIMAKE. Protein marker (26616) was purchased from Thermo Fisher Scientific. RIPA Lysis Buffer (P0013B), Cell Mitochondria Isolation Kit and 5× loading buffer of SDS-PAGE were purchased from Beyotime. Fetal bovine serum (10100147C), 1640 medium (11875093), and DMEM medium (C11995500BT) were purchased from Gibco. ROS detection assay kit (E004-1-1) was purchased from NJJCBio (Nanjing, China).

### Cell culture and transfection

The human ovarian cancer cell lines A2780, SkOV3, CaoV3, and 293T cells were purchased from National Collection of Authenticated Cell Cultures (Shanghai, China). IOSE-80 cells were purchased from Cellverse (Shanghai, China). A2780, SkOV3, CaoV3, and 293T cells were cultured in DMEM medium supplemented with 10% FBS at 37 °C in a 5% CO_2_ atmosphere. IOSE-80 cells were cultured in 1640 medium under the same conditions. Plasmids were transfected into cells using lipofectamine 2000.

### CCK8 assay

A total of 5000 cells were seeded in each well of a 96-well plate. After 12 h of incubation, the cells were treated with different concentrations of cisplatin, with three biological replicates for each group. The absorbance at 450 nm was measured using the CCK8 kit (BIOGROUND). The cisplatin IC50 value was calculated using GraphPad Prism 9 software.

### Colony formation and Transwell assays

For the colony formation assay, 500 cells were seeded in 6-well plates with a complete medium. After incubation for 7–14 days, the cells were stained with 0.01% crystal violet at room temperature (RT) for 1 h.

For the Transwell assay, either collagen I-coated (60 µg/Transwell) membranes for the invasion assay or uncoated membranes for the migration assay were placed in the upper chamber. The lower chamber contained 0.65 mL of medium. 10^5^ cells were seeded in the upper chamber and incubated for 24–48 h. Non-migrated cells in the upper chamber were removed by scraping with a cotton swab. The upper chambers were then immersed in 4% PFA for 15 min at room temperature, rinsed twice with PBS, and stained with 0.25% crystal violet for 30 min at room temperature. After rinsing twice with PBS, the upper chambers were allowed to air-dry. The total number of migrated or invaded cells was counted using a fluorescence microscope (NIKON, Ts2R).

### RNA interference

For transient knock down of endogenous genes expression, siRNAs were transfected into the target cell line by lipofectamine 2000 according to the manufacturer’s protocol (Thermo Fisher Scientific). Briefly, the cells were collected 48 h after siRNA transfection, and then the knockdown efficiency of the target gene was confirmed by real-time quantitative PCR and Western blotting. To achieve stable knockdown of target genes, shRNA and lentivirus packaging plasmids were co-transfected into 293T cells. After 72 h, the supernatant was harvested, cleared of cell debris by centrifugation at 4 °C, and used for transfection of the target cell lines. Knockdown efficiency was assessed 72 h after transfection. The target sequences of the indicated genes are list as below:

siAkt1: 5′- CCAAGGAGAUCAUGCAGCA-3′, siAkt2: 5′- CGACUGAGGAGAUGGAAGU-3′; siAkt3 5′- ACCAGAGGUGUUAGAAGA-3′

### Immunoblots and immunoprecipitation

For western blotting, cells were lysed in RIPA buffer (50 mM Tris(pH 7.4), 150 mM NaCl, 1% Triton X-100, 1% sodium deoxycholate, and 0.1% SDS) containing protease inhibitors Cocktail (BIMAKE). Then, the samples with SDS loading buffer were boiled at 95 °C for 10 min, separated by SDS-PAGE, transferred onto polyvinylidene difluoride (PVDF) membrane (Millipore), incubated with specific primary and secondary antibodies at 4 °C overnight, and detected with Super ECL Plus Western Blotting Substrate (BIOGROUND) in the ChemiDoc XRS+ Gel Imaging System (Bio-Rad).

For immunoprecipitation, cells were lysed in IP buffer (20 mM Tris(pH7.5), 150 mM NaCl, 1% Triton X-100, and 1 mM EDTA) supplemented with protease inhibitors Cocktail and clarified by centrifugation. Subsequently, the supernatant was incubated with indicated primary antibody for 3–4 at 4 °C followed by protein A Sepharose beads (Bayotime) Following thorough washing, both the immunoprecipitated protein and whole cell lysis underwent western blot analysis.

### Lactate dehydrogenase (LDH) release assay

LDH produced within cells was detected using an LDH release Assay kit (Promega). Briefly, the cells were subjected to treatment with DDP along with either specific inhibitors or without inhibitors. Subsequently, the cell supernatant was collected after centrifugation at 400 rpm for 5 min at 4 °C and used for LDH detection following the manufacturer’s protocol. The absorbance of each well was measured at the wavelength of 490 nm using Infinite 2000 Pro Microplate reader (TECAN).

### ROS detection assay

Ovarian cancer cells were seeded at 5 × 10^3^ cells per well in a 96-well plate and allowed to adhere overnight. Subsequently, cells were treated with or without cisplatin at its IC50 concentration for 24 h. Following treatment, cells were rinsed with pre-warmed DMEM medium and then exposed to 10 μM DCFH-DA working solution for 30 min at 37 °C in darkness. After incubation, cells were washed with PBS to remove excess DCFH-DA. Fluorescence intensity indicative of ROS levels was measured using a microplate reader set at excitation/emission wavelengths of 488/525 nm.

### Reverse transcription-quantitaitive PCR(RT-qPCR)

Total RNA from ovarian cancer cell lines was extracted using TRIzol® reagent (Thermo Fisher Scientific) according to the manufacturer’s protocol. Briefly, Ovarian cancer cells were collected in a 2 ml tube containing 1 ml Trizol. After vortexing for 2 min, the mixture was centrifuged at 12,000 × *g* for 5 min at 4 °C. The supernatant was transferred to a new 1.5 ml EP tube with 0.2 ml chloroform alcohol. Following vigorous shaking, the mixture was centrifuged again at 12,000 × *g* for 10 min at 4 °C. The upper aqueous phase was transferred to a new tube with an equal volume of isopropyl alcohol and centrifuged at 12,000 × *g* for 10 min at 4 °C. The supernatant was discarded, and the RNA pellet was washed twice with 1 ml 75% ethanol. After centrifugation at 12,000 × *g* for 5 min at 4 °C, the pellet was air-dried for 5–10 min in the biosafety cabinet. Finally, 20 μl of RNA-free water was added to dissolve the RNA, and the total RNA was subsequently qualified.

Total RNA was reverse transcribed into cDNA using the PrimeScript™ RT reagent Kit (TAKARA). Real-time was performed using TB Green™ Premix ExTaq™ (TAKARA) in a Bio-Rad CFX96 system. The listed below are PCR primers used in this study: RT-Actin-F: 5′-CAAAGTTCACAATGTGGCCGAGGA-3′, RT-Actin-R: 5′-GGGACTTCCTGTAACAACGCATCT-3′; RT-CNTFR-F: 5′-CTGGGCTCTGACGTGACAC-3′, RT-CNTFR-R: 5′- GTGGAAGCAGGCGTAGAGG-3′; RT-CLCF1-F: 5′-TTTCAACGAGCCAGACTTCAAC-3′, RT-CLCF1-R: 5′-GAGGCCACGCAAGTAACACA-3′; RT-CRLF1-F: 5′-CTCTCCCGTGTACTCAACGC-3′, RT-CRLF1-R: 5′-GGGCAGGCCAACATAGAGG-3′; RT-caspase3-F: 5′-TGGCGAAATTCAAAGGATG-3′, RT-caspase3-R: 5′-TGGCGAAATTCAAAGGATG-3′.

### Total mRNA sequencing

Total RNA from ovarian cancer control cells and cells overexpressing CRFL1, with or without DDP treatment, was extracted using TRIzol® reagent (Thermo Fisher Scientific) following the manufacturer’s protocol. Reverse transcription was performed using SuperScript II reverse transcriptase (Thermo Fisher Scientific, 18064-014). The PCR program included incubation at 42 °C for 90 min, followed by 10 cycles of 50 °C for 2 min, 42 °C for 2 min, 70 °C for 15 min, and a final hold at 4 °C. The primers used were: 3’ CDS primer (5’-AAGCAGTGGTATCAACGCAGAGTACT30VN-3’) and LNA-TSO primer (5’-AAGCAGTGGTATCAACGCAGAGTACATrGrG+G-3’), where ‘N’ represents any base, ‘V’ represents A, C, or G, ‘rG’ represents riboguanosine, and ‘+G’ represents LNA-modified guanosine. The PCR products were purified using Ampure XP Beads, and DNA concentration was measured using the Equalbit® 1 × dsDNA HS Assay Kit (Vazyme, EQ121). Subsequently, 50 ng of purified cDNA library was digested at 55 °C for 5 min with transposase (TruePrepTM DNA Library Prep Kit V2 for Illumina®, Vazyme #TD501). The DNA fragments were then purified using Ampure XP Beads. Purified DNA fragments were ligated with N7 and N5 adaptors containing various indicators using the TruePrepTM Index Kit V2/V3 for Illumina® (Vazyme #TD202), as per the manufacturer’s protocol. Sequencing was performed on an Illumina Hiseq 4000 machine. RNA expression abundance was determined based on FPKM (fragments per kilobase of transcript per million mapped reads) calculated using rsem-calculate-expression. The top 10% of differential expressed genes with FPKM > 5 in all three biological duplicate samples were selected for further GO and KEGG pathway analysis.

### Mitochondrial isolation

Mitochondria were isolated according to the manufacturer’s protocol. Briefly, cells were enzymatically dissociated using trypsin-EDTA and isolated by centrifugation. Subsequently, the cells were washed with PBS, resuspended in mitochondrial isolation medium, and homogenized. The homogenate was centrifuged to obtain a mitochondrial fraction, which was then purified through additional centrifugation to eliminate residual supernatant. The resultant mitochondrial pellet was utilized for subsequent protein assays.

### Ethics statement

ALL animal procedures were conducted in accordance with the guidelines and approval of the Women and Children’s Hospital of Chongqing Medical University.

### Tumor xenograft model

Female null mice, 6 weeks old, were subcutaneously implanted with either 1.5 × 10^6^ contorl cells or 1.5 × 10^6^ CaoV3 cells overexpressing CRLF1, suspended in a 1:1 mixture of DMEM medium and matrix gel. Once tumors were observed, mice were randomly assigned to three groups (*n* = 5/group): mimic group, DDP-treated group, and DDP + MK2206 treatment group. The treatment consisted of intraperitoneal injection of 4 mg/kg DDP and intragastric administration of MK2206, with a frequency of three times per week. After two and a half weeks, mice were euthanized using CO_2_ inhalation, and tumor tissues were excised. Then, tumor weight, imaging, and volume were determined. Additionally, 50 mg of tumor tissues were lysed in RIPA buffer supplemented with Proteinase Cocktail for western blotting. Tumor volumes were calculated using the formula: length × width^2^/2.

### Quantification and statistical analysis

Statistical data are presented as mean values ± SEM or SD and analyzed using GraphPad Prism V9.0 (San Diego, CA). Two-tailed Student’s *t*-test was used to compare continuous variables between two groups, while one-way/two-way ANOVA was utilized for comparisons involving three or more groups. Quantitative data from cellular experiments were derived from a minimum of three independent biological replicates. Except for mice tumor xenograft assays, no data point was excluded. For tumor xenograft assays, the top and bottom values were discarded before calculating the average.

## Supplementary information

Original western blots

Supplementary Figures

Data S1

## Data Availability

This study includes no data deposited in external repositories. All data and materials can be found in the main text and supplementary materials. For requests regarding primary datasets and materials, please contact XWC.
