# Supplementary figures and images for: CRLF1 bridges AKT and mTORC2 through SIN1 to inhibit pyroptosis and enhance chemo-resistance in ovarian cancer
Source: Cell Death Dis. 2024 Sep 10;15(9):662. doi: 10.1038/s41419-024-07035-4 (PMC11387770; doi:10.1038/s41419-024-07035-4)

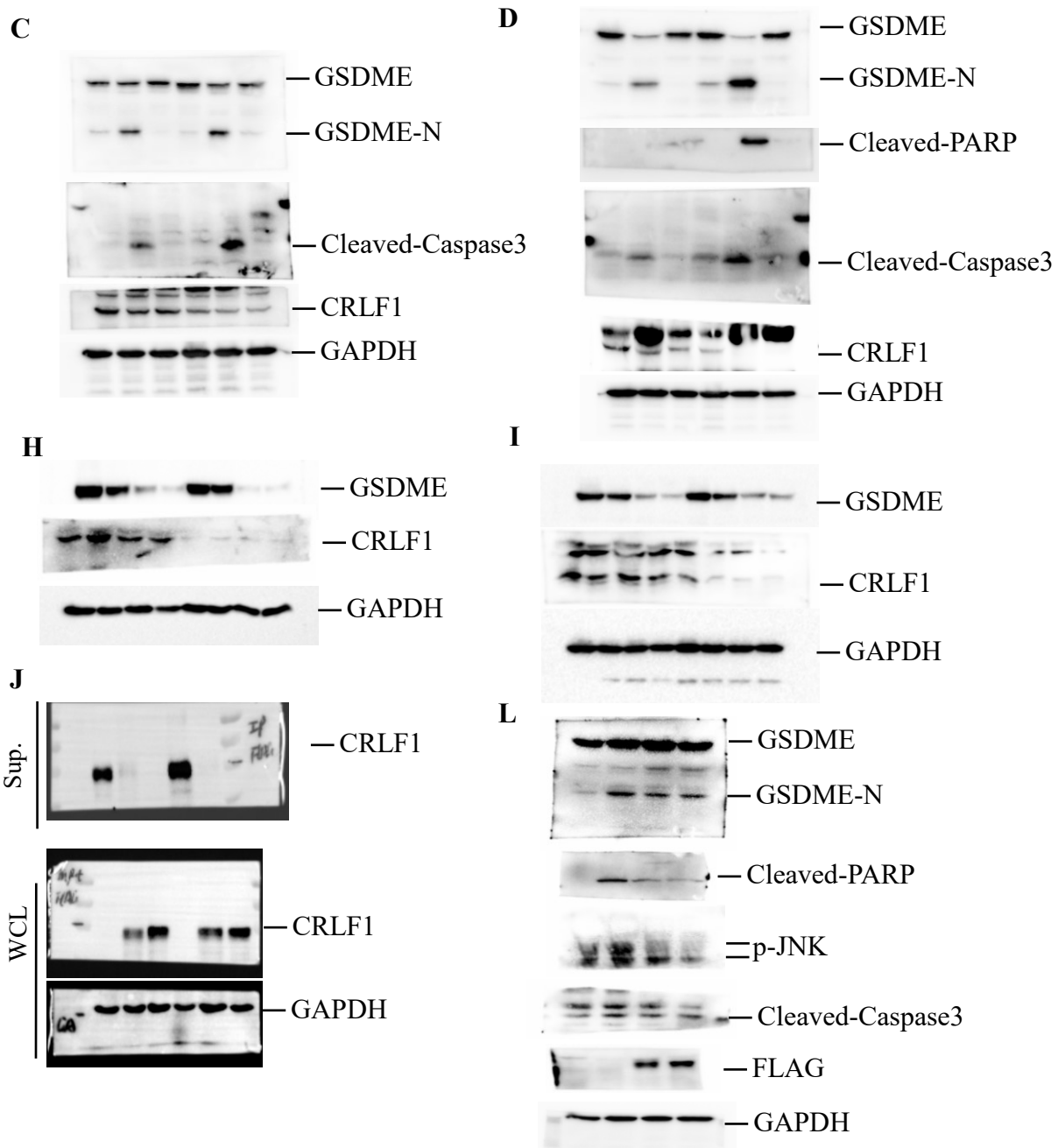

**Fig. 3**

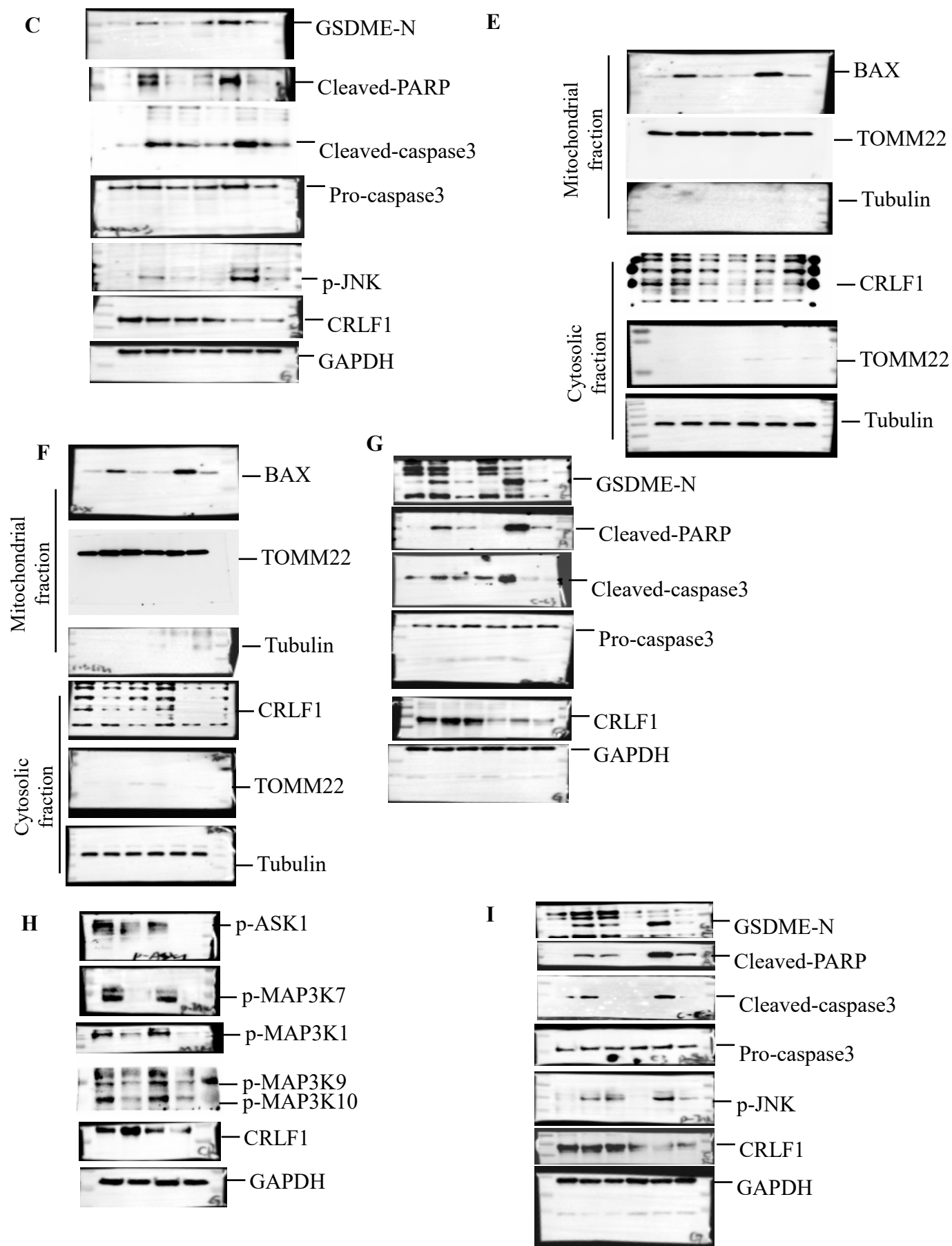

**Fig. 4**

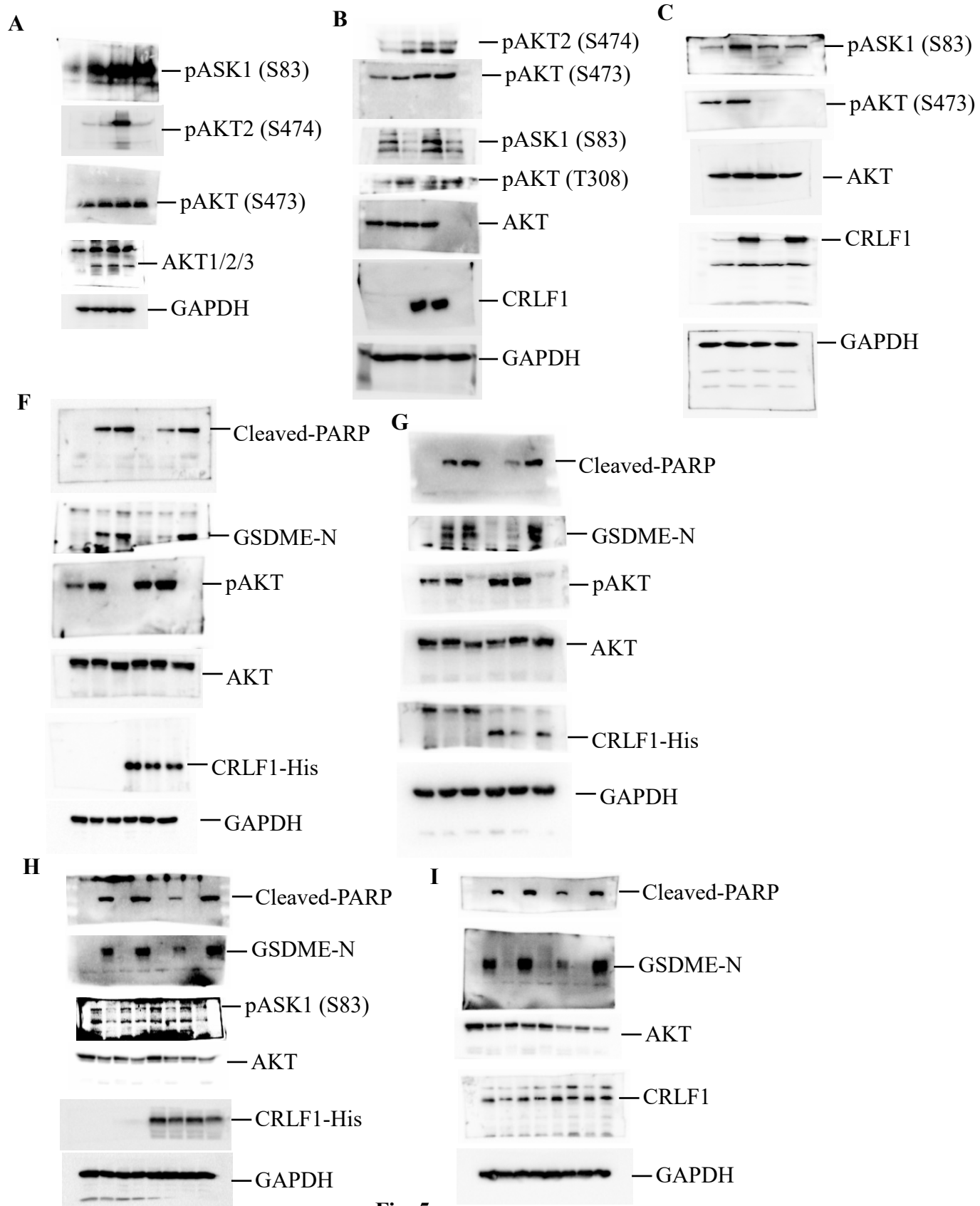

**Fig. 5**

**E**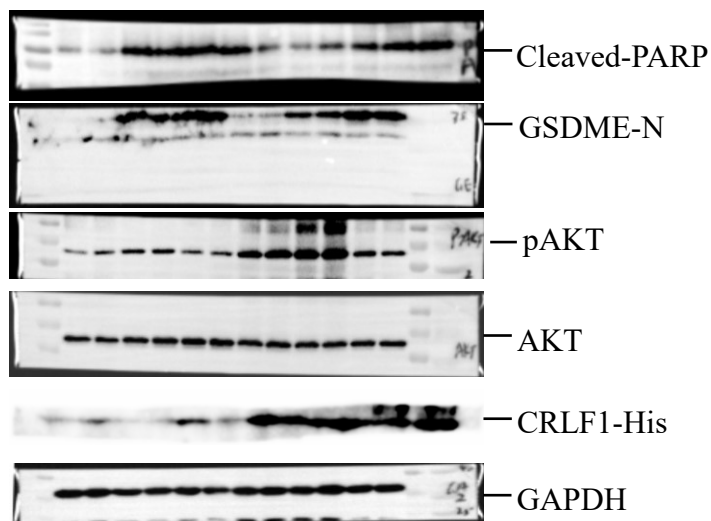**F**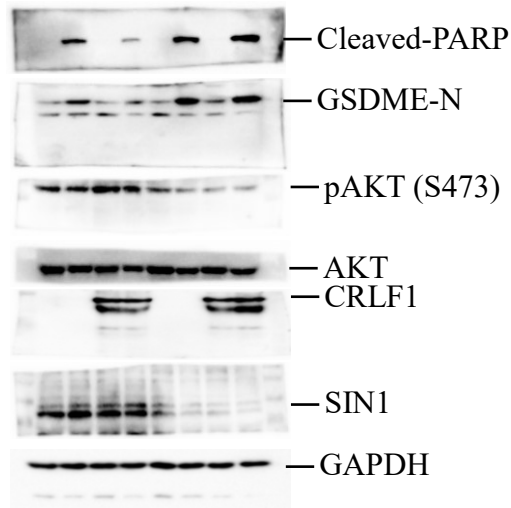**Fig. 6****A**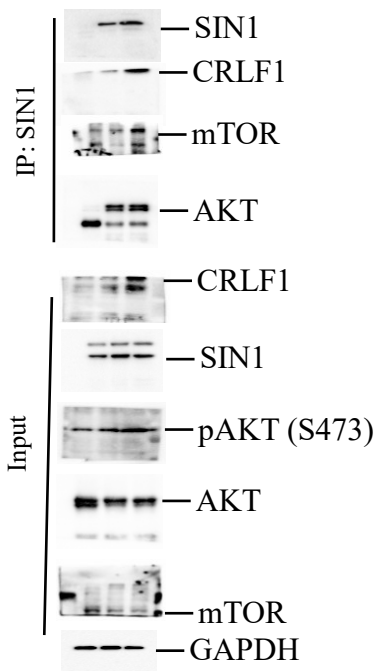**B**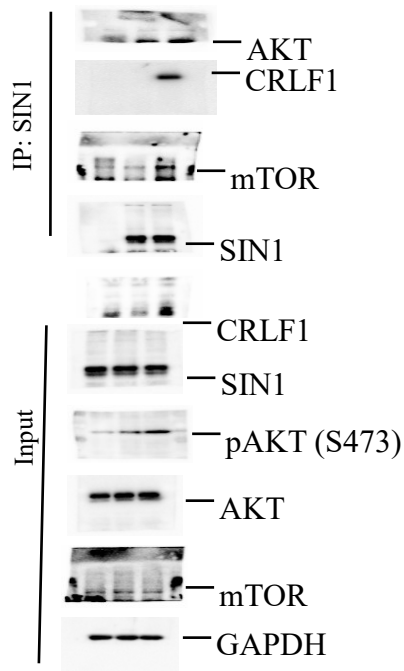**C**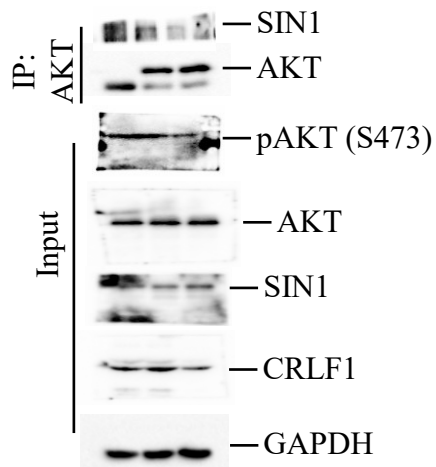**H**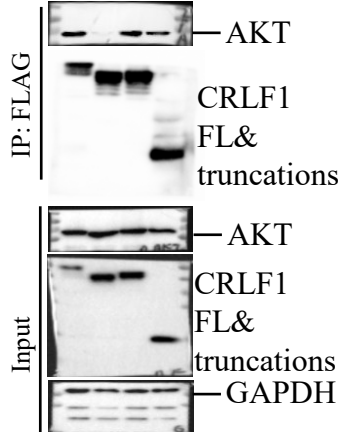**I**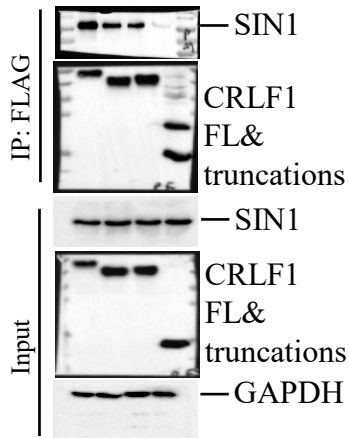**J**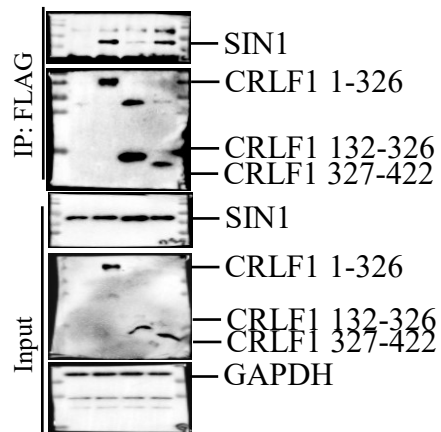**Fig. 7**

**C**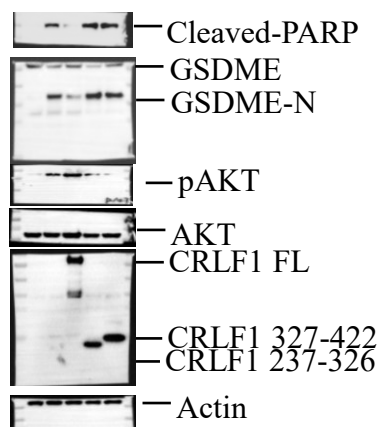**D**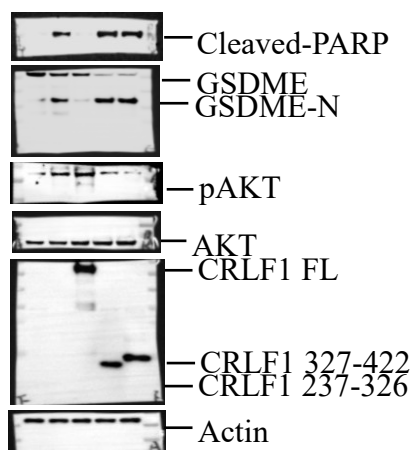**E**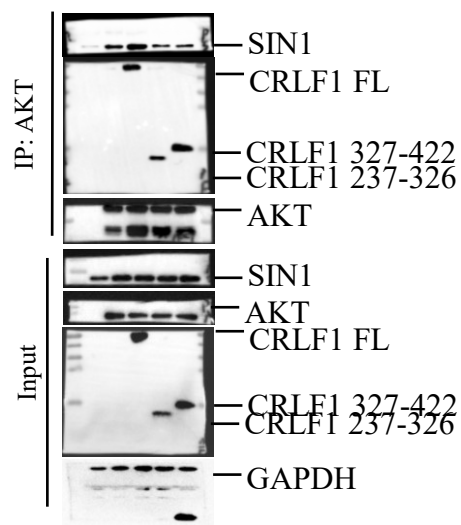**F**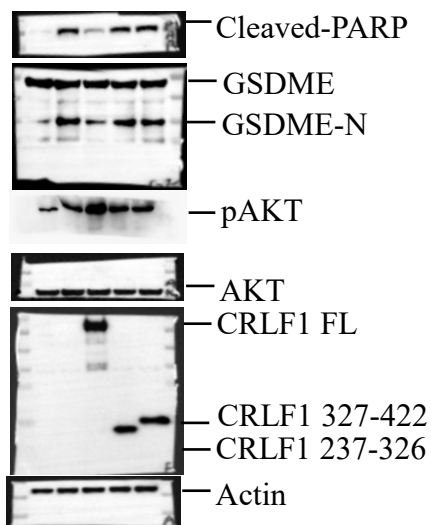**Fig. 8****C**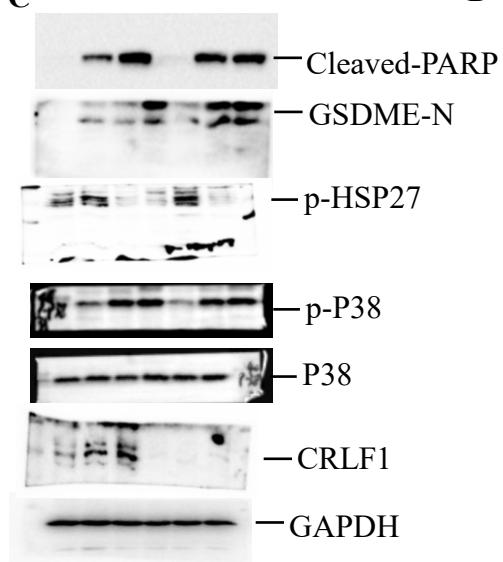**D**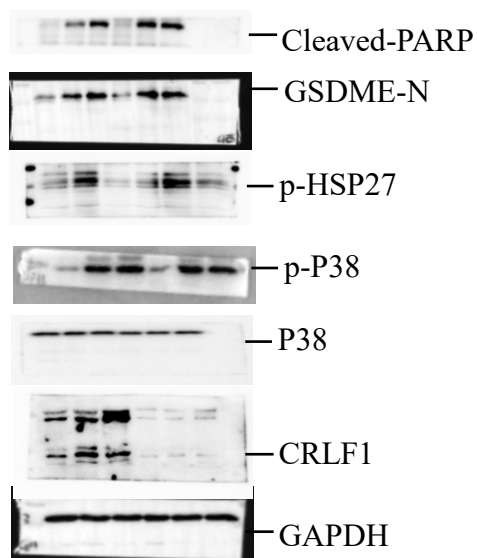**Fig. S4**

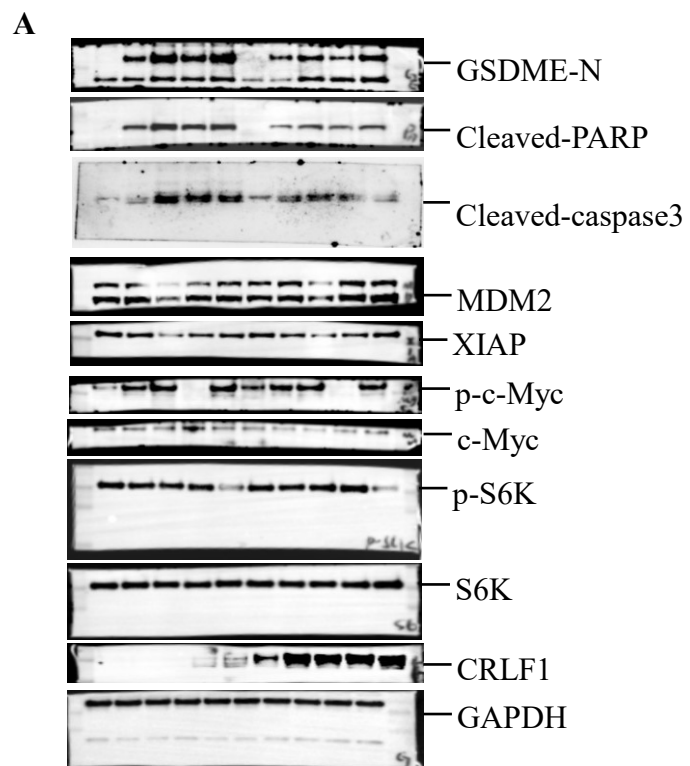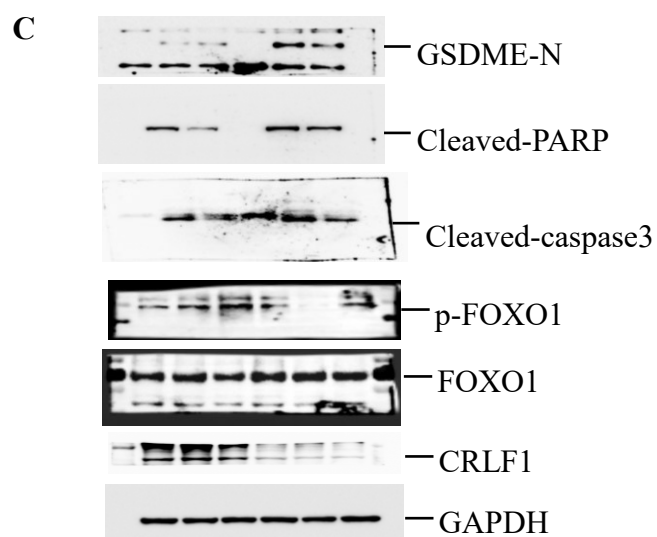

**Fig. S6**

A

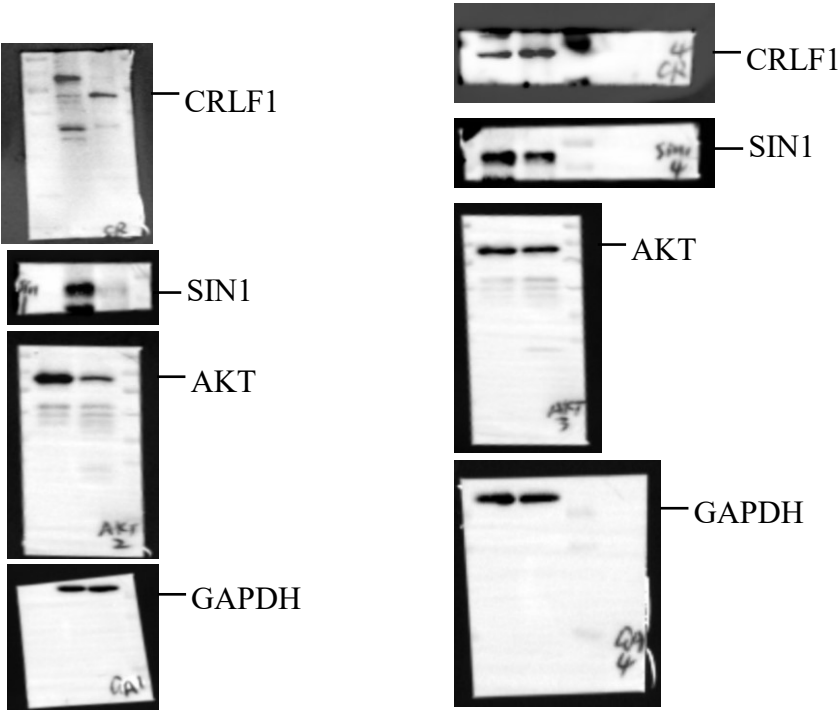

E

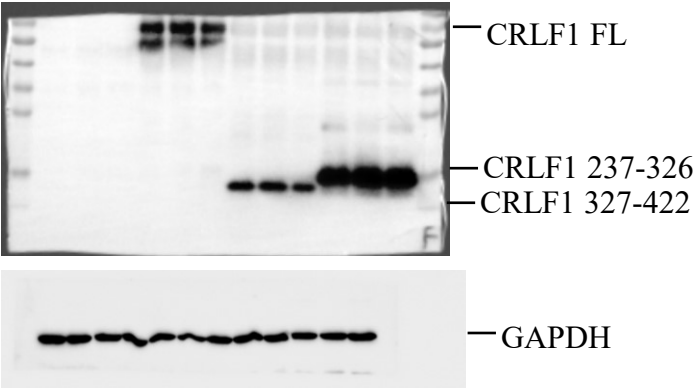

Fig. S7

Supplement: Supplementary file 1 — Original western blots [file 41419_2024_7035_MOESM1_ESM.pdf]
