# Supplementary material for: CRLF1 bridges AKT and mTORC2 through SIN1 to inhibit pyroptosis and enhance chemo-resistance in ovarian cancer
Source: Cell Death Dis. 2024 Sep 10;15(9):662. doi: 10.1038/s41419-024-07035-4 (PMC11387770; doi:10.1038/s41419-024-07035-4)
Supplement: Supplementary file 2 — Supplementary Figures [file 41419_2024_7035_MOESM2_ESM.pdf]

Supplementary Figures

Fig. S1.

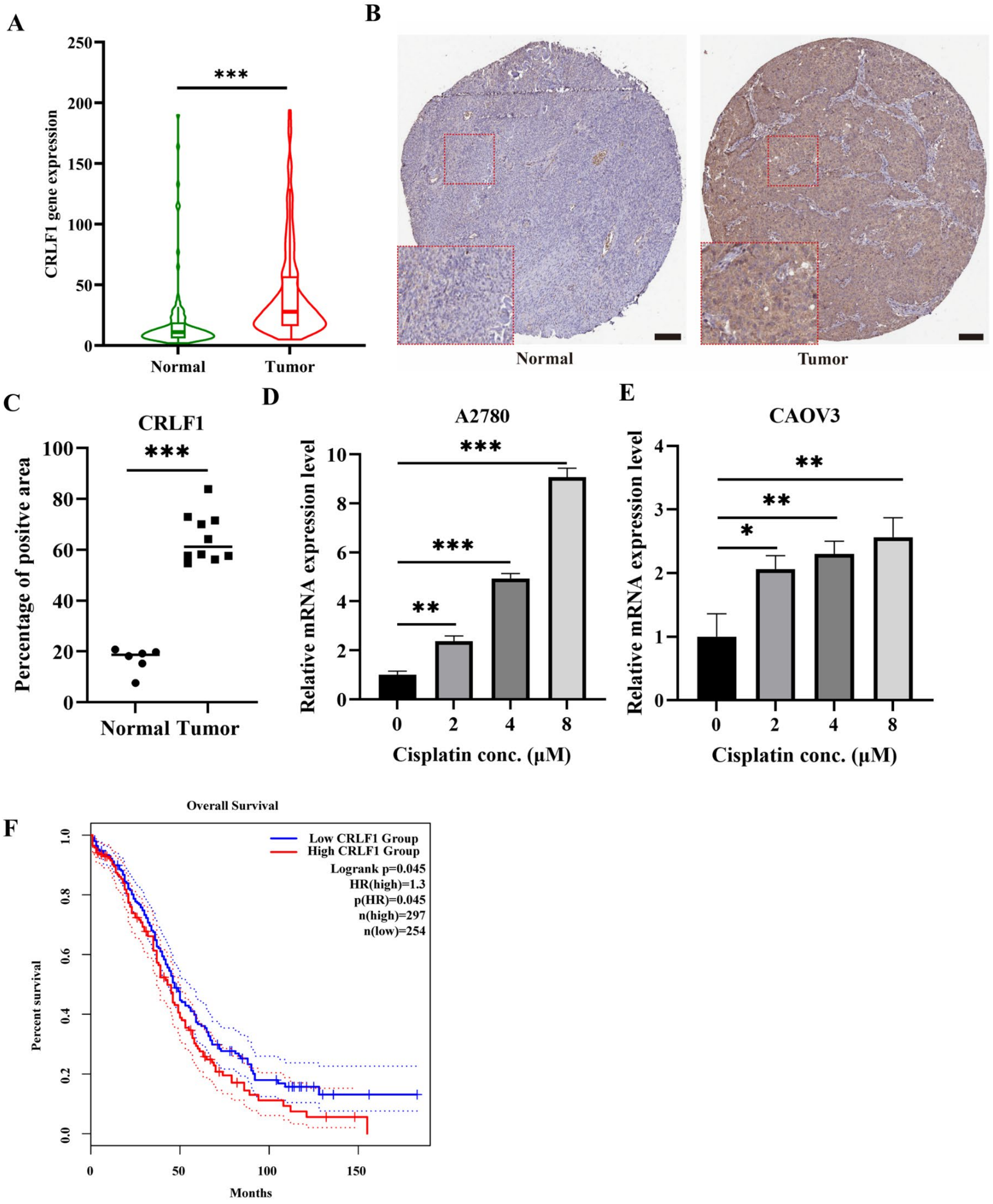

Fig. S1. CRLF1 expression is increased in ovarian cancer tissues and cisplatin treatment further increases its levels. **(A).** TGCA database analysis demonstrated that CRLF1 expression was upregulated in ovarian cancer. **(B)**. The protein levels of CRLF1 in both normal and tumor ovarian tissues were assessed using immunohistochemistry (IHC) results obtained from the Human Protein Atlas (HPA). The scale bar is set at 100 μm for the images. **(C).** Quantification of

CRLF1 expression levels in **(B)** using Image J software. (**D&E).** Cisplatin upregulated CRLF expression in A2780 cells **(D)** and CaoV3 cells **(E).** After treated with cisplatin at the indicated time points, CRLF1 mRNA levels in A2780 cells and CaoV3 cells were quantified by Real-time Quantitative PCR (qPCR). **F.** High CRLF1 level predicts shorter overall survival (OS) by online GEPIA2 analysis. For graphs, error bars are mean ± s.e.m. *P* values were determined by one-way ANOVA or unpaired *t* test (two-tailed), *p<0.05, **p<0.01, ***p<0.001.

Fig. S2.

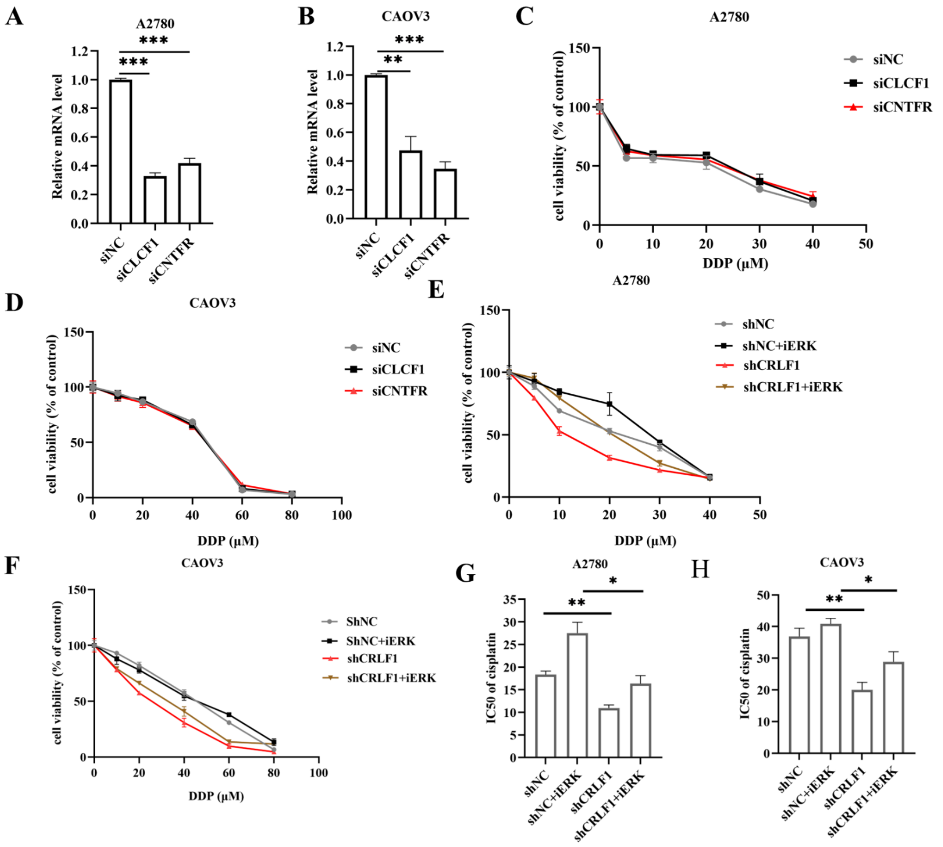

Fig. S2. CRLF1 enhances chemoresistance independently of GP130/JAK signaling pathway. **(A&B).** Knockdown of CLCF1 or CNTFR in A2780 cells **(A)** and CaoV3 cells **(B)**, mRNA level evaluated by qPCR. **(C&D).** A2780 cells **(C)** and CaoV3 cells **(D)** were treated with the indicated dose of cisplatin for 24h. Cell viability was assessed by CCK-8 assay. **(E&F).** A2780 cells **(E)** and CaoV3 cells **(F)** were treated with ERK inhibitor, followed by IC50 dose of cisplatin treatment. Cell viability was measured by CCK-8 assay. **(G&H).** Cisplatin IC50 of A2780 cells **(G)** and CaoV3 cells **(H)** were calculated on GraphPad Prism 9. For all graphs, error bars represent mean ± s.e.m. *P* values were determined by one-way ANOVA unless otherwise indicated, *p<0.05, ***p<0.001.

Fig. S3.

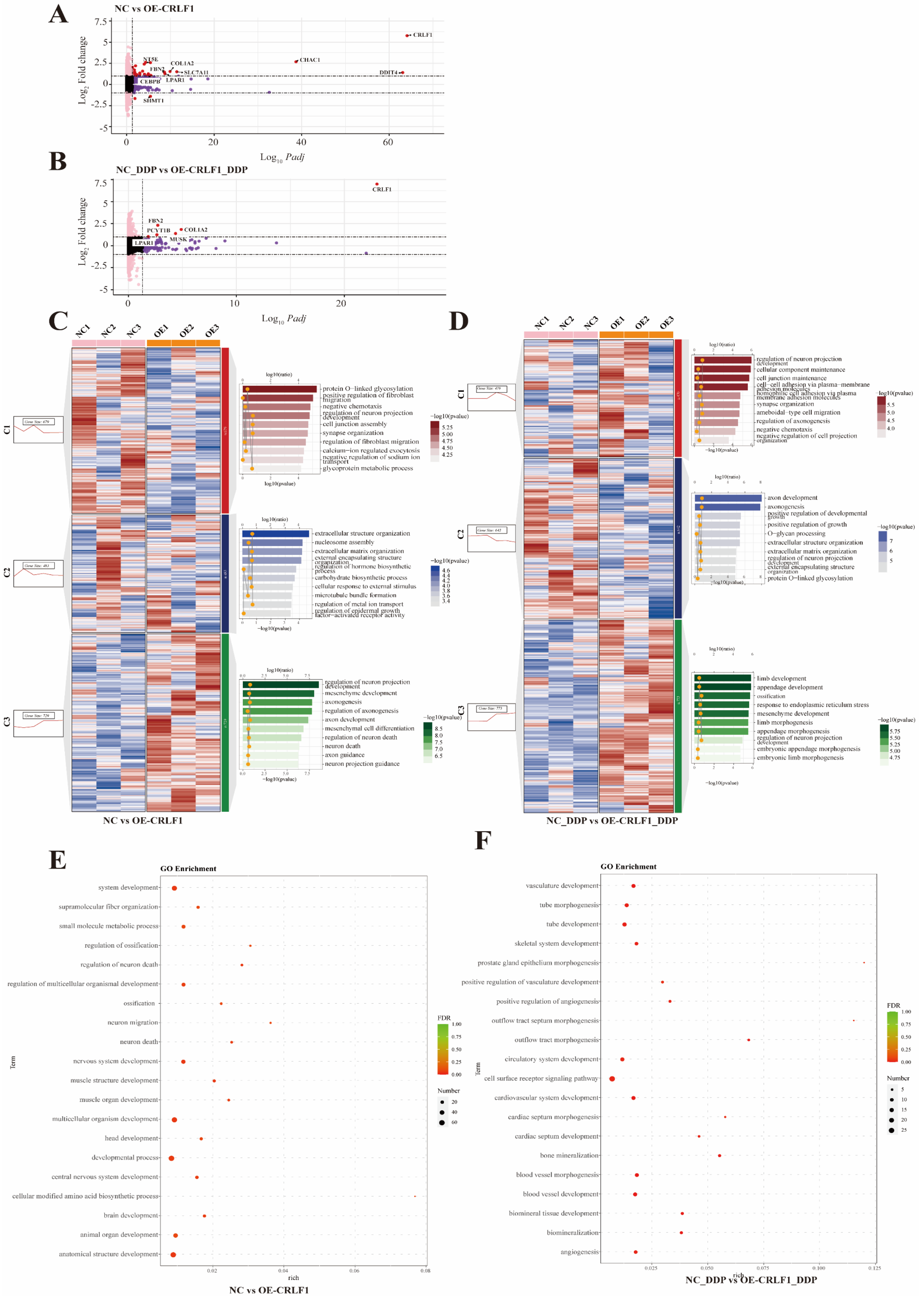

A
NC vs OE-CRLF1
CRLF1
CHAC1
DDIT4
NT5E
COL1A2
FBN2
SLC7A11
CEBPB
LPAR1
SHMT1
B
NC_DDP vs OE-CRLF1_DDP
CRLF1
FBN2
PCYT1B
COL1A2
MUSK
LPAR1
C
NC1
NC2
NC3
OE1
OE2
OE3
C1
C2
C3
protein O−linked glycosylation
positive regulation of fibroblast migration
negative chemotaxis
regulation of neuron projection development
cell junction assembly
synapse organization
regulation of fibroblast migration
calcium−ion regulated exocytosis
negative regulation of sodium ion transport
glycoprotein metabolic process
extracellular structure organization
nucleosome assembly
extracellular matrix organization
external encapsulating structure organization
regulation of hormone biosynthetic process
carbohydrate biosynthetic process
cellular response to external stimulus
microtubule bundle formation
regulation of metal ion transport
regulation of epidermal growth factor−activated receptor activity
regulation of neuron projection development
mesenchyme development
axonogenesis
regulation of axonogenesis
axon development
mesenchymal cell differentiation
regulation of neuron death
neuron death
axon guidance
neuron projection guidance
log10(ratio)
−log10(pvalue)
NC vs OE-CRLF1
D
regulation of neuron projection development
cellular component maintenance
cell junction maintenance
cell−cell adhesion via plasma−membrane adhesion molecules
homophilic cell adhesion via plasma membrane adhesion molecules
synapse organization
ameboidal−type cell migration
regulation of axonogenesis
negative chemotaxis
negative regulation of cell projection organization
axon development
axonogenesis
positive regulation of developmental growth
positive regulation of growth
O−glycan processing
extracellular structure organization
extracellular matrix organization
regulation of neuron projection development
external encapsulating structure organization
protein O−linked glycosylation
limb development
appendage development
ossification
response to endoplasmic reticulum stress
mesenchyme development
limb morphogenesis
appendage morphogenesis
regulation of neuron projection development
embryonic appendage morphogenesis
embryonic limb morphogenesis
NC_DDP vs OE-CRLF1_DDP
E
GO Enrichment
system development
supramolecular fiber organization
small molecule metabolic process
regulation of ossification
regulation of neuron death
regulation of multicellular organismal development
ossification
neuron migration
neuron death
nervous system development
muscle structure development
muscle organ development
multicellular organism development
head development
developmental process
central nervous system development
cellular modified amino acid biosynthetic process
brain development
animal organ development
anatomical structure development
Term
rich
FDR
Number
NC vs OE-CRLF1
F
GO Enrichment
vasculature development
tube morphogenesis
tube development
skeletal system development
prostate gland epithelium morphogenesis
positive regulation of vasculature development
positive regulation of angiogenesis
outflow tract septum morphogenesis
outflow tract morphogenesis
circulatory system development
cell surface receptor signaling pathway
cardiovascular system development
cardiac septum morphogenesis
cardiac septum development
bone mineralization
blood vessel morphogenesis
blood vessel development
biomineral tissue development
biomineralization
angiogenesis
NC_DDP vs OE-CRLF1_DDP

Fig. S3. The regulatory roles of CRLF1 vary depending on the context. **(A&B).** Volcano plots were used to display the top 10 differentially expressed genes in the non-DDP treatment group **(B)** and genes with Log2 (fold change)>1 in the DDP treatment group **(A). (C&D).** Cluster analysis of differentially expressed genes in the DDP-treated **(D)** or untreated group **(C)**. Genes with similarly expressing only found in Cluster3, these genes were upregulated by CRLF1 in two groups. unsupervised hierarchical clustering was performed using Clustal 3. (**E&F).** The top twenty GO terms in the DDP treatment group **(F)** and non-DDP treatment group **(E)** were presented. The size of the circles indicates the number of differentially expressed genes (DGEs) and the color of the circle represents *p* value.

Fig. S4.

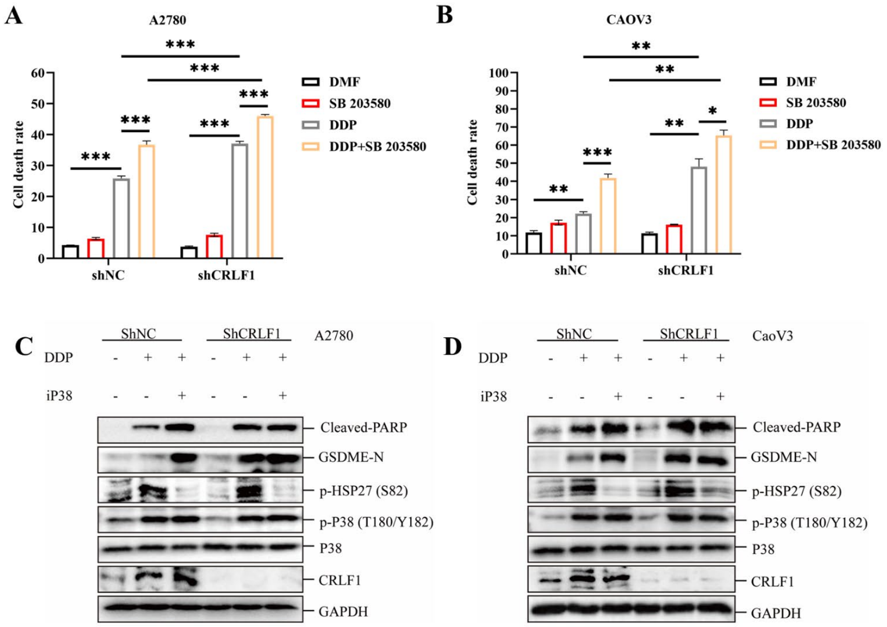

Fig. S4. **CRLF1 dampens pyroptosis regardless of p38 activity.** Half an hour after 10 µM P38 inhibitor was added, the indicated cell lines were treated with DDP for 18h. LDH assay **(A and B)** and western blotting were employed to evaluate the cell death of A2780 cells and CaoV3 cells **(C and D)**, respectively. For all graphs, error bars represent mean ± s.e.m. *P* values were determined by two-way ANOVA, *p<0.05, ***p<0.001.

Fig. S5.

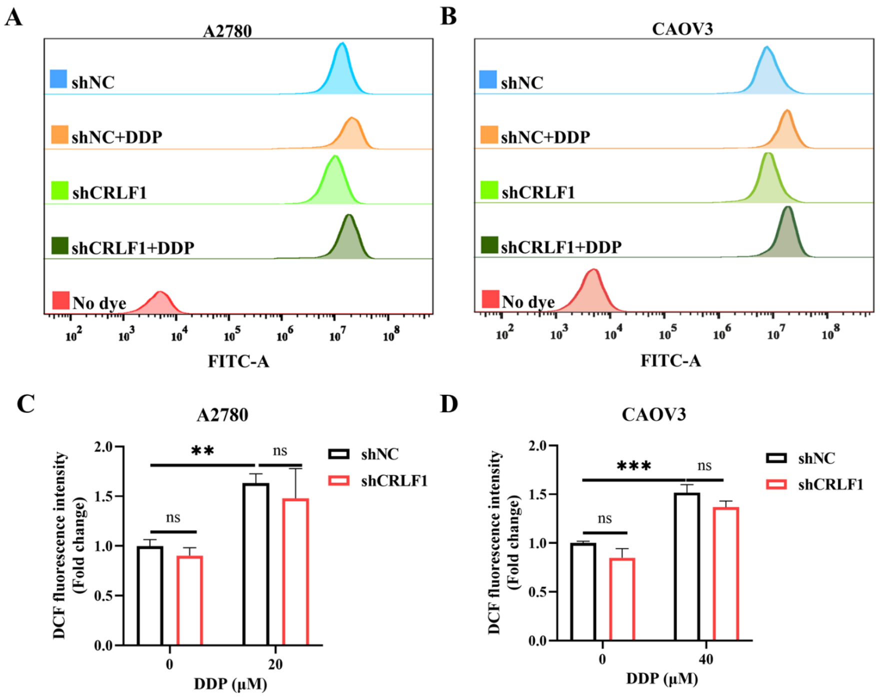

Fig. S5. CRLF1 has no effect on ROS production. **(A&B)**. A2780 cells (**A**) and CaoV3 cells (**B**) were stained with 20 μM DCFDA, a fluorogenic dye, for 45 min at 37°C to measure intracellular ROS. After staining, cells were collected and analyzed on flow cytometer at 485 and 535 nm of maximum excitation and emission spectra, respectively. **(C&D).** Quantification of intracellular ROS in A2780 cells (**C**) and CaoV3 cells (**D**). For all graphs, error bars represent mean ± s.e.m. *P* values were determined by one-way ANOVA, *p<0.05, ***p<0.001.

Fig. S6

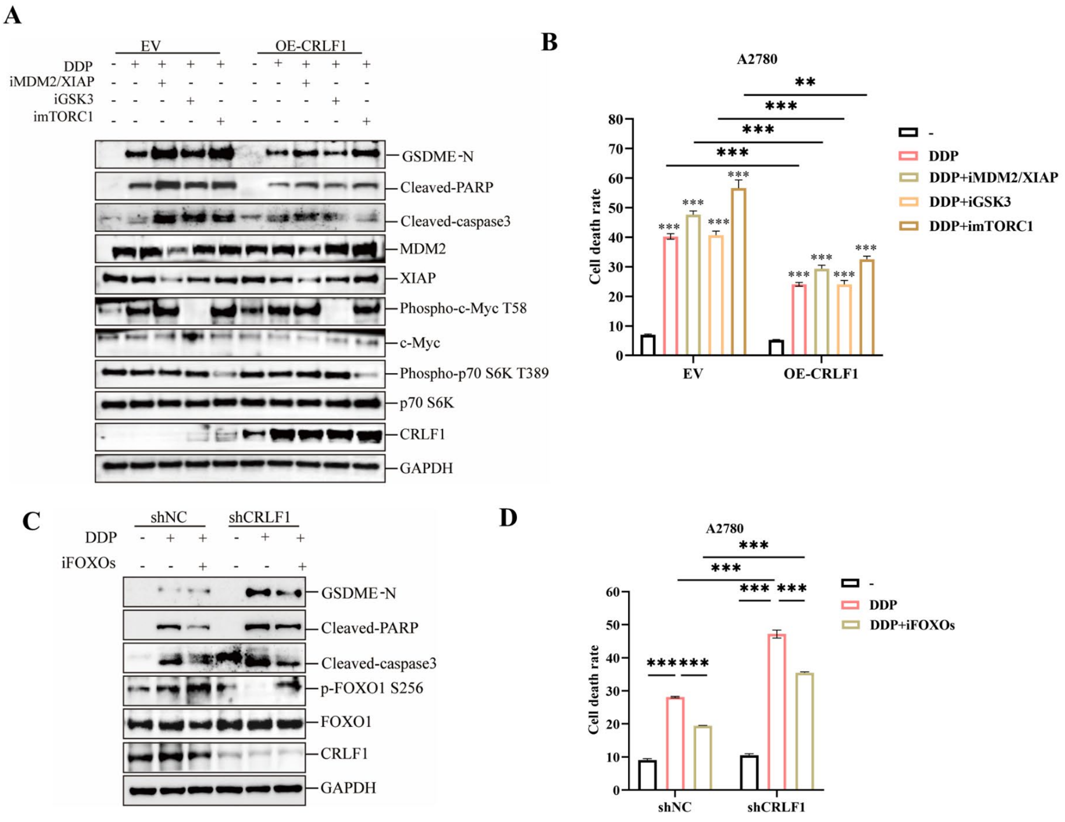

Fig. S6. Inhibition of MDM2/XIAP/GSK3/FOXO1/mTORC1 failed to counteract CRLF1's modulation of DDP-evoked pyroptosis. **(A&B)**. Caov3 cells were transfected with CRLF1 overexpression plasmids or control plasmids. The cells were then treated with the indicated inhibitors in combination with DDP to evaluate cell death using western blotting **(A)** and LDH release assay **(B)**. **(C&D).** Cells with CRLF1 knockdown and control cells were exposed to DDP in the presence or absence of FOXOs inhibitor. Cell death was evaluated by western blotting **(C)** and LDH release assay **(D)**.

Fig. S7

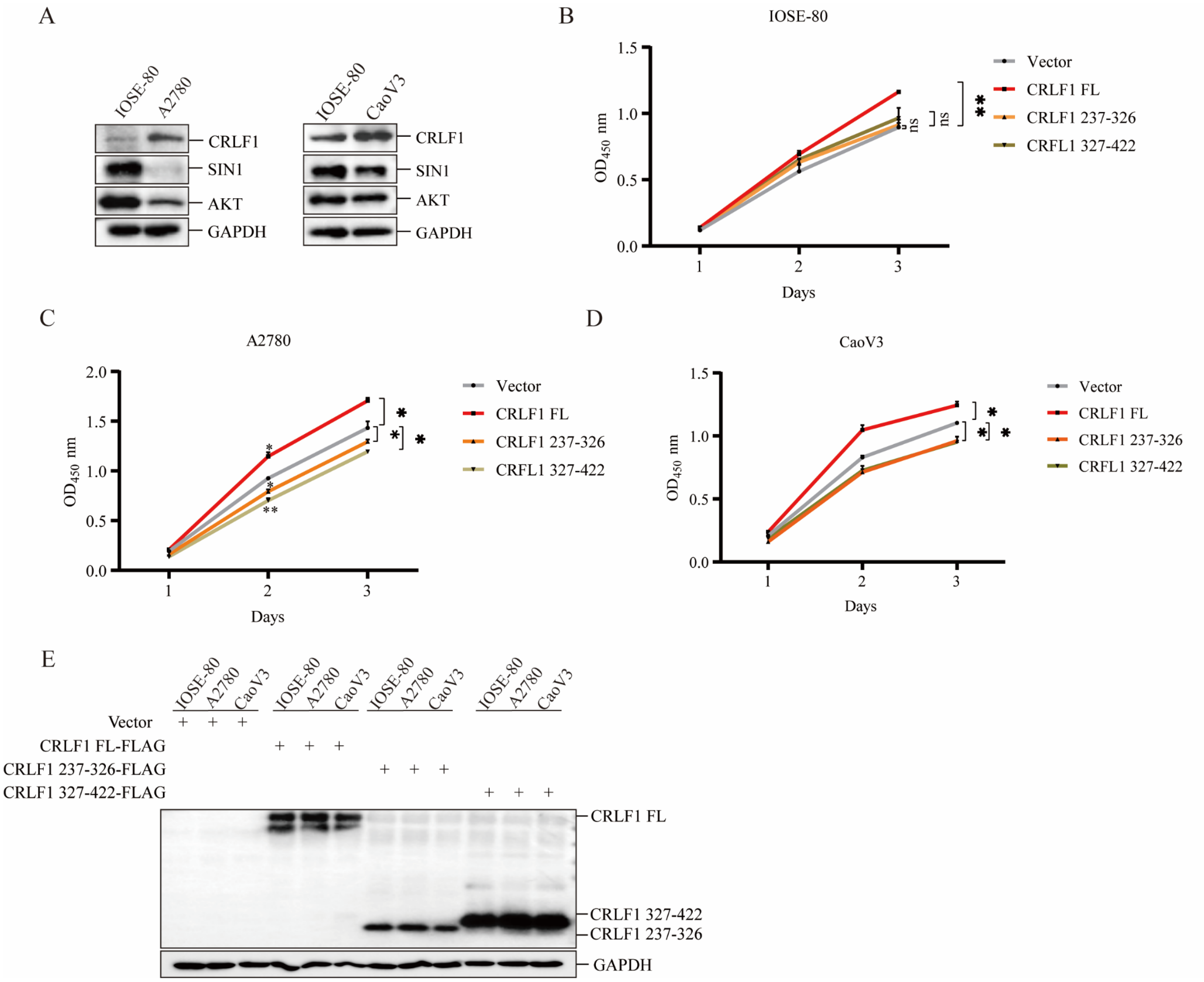

Fig. S7. Overexpression of binding-defective CRLF1 variants specifically inhibits tumor cell proliferation. (A). The CRLF1 expression levels in IOSE-80, A2780, and CaoV3 cells were detected by western blotting. **(B-D)**. Cell proliferation in IOSE-80 **(B)**, A2780 **(C)**, and CaoV3 cells **(D)** was assessed by CCK-8 assay after transfection with the indicated plasmids. **(E).** The protein expression levels of the indicated plasmids were assessed using western blotting. For graphs, error bars represent mean ± s.e.m. *P* values were determined by one-way ANOVA, *p<0.05, ***p<0.001.
